# Sources of inter-individual variability leading to significant changes in anti-PD-1 and anti-PD-L1 efficacy identified in mouse tumor models using a QSP framework

**DOI:** 10.3389/fphar.2022.1056365

**Published:** 2022-12-05

**Authors:** Jessica C. Leete, Michael G. Zager, Cynthia J. Musante, Blerta Shtylla, Wenlian Qiao

**Affiliations:** ^1^ Clinical Pharmacology, Early Clinical Development, Pfizer Inc., Cambridge, MA, United States; ^2^ Translational Modeling and Simulation, BioMedicine Design, Pfizer Inc., Cambridge, MA, United States; ^3^ Translational Modeling and Simulation, BioMedicine Design, Pfizer Inc., La Jolla, CA, United States; ^4^ Quantitative Systems Pharmacology, Early Clinical Development, Pfizer Inc., Cambridge, MA, United States; ^5^ Quantitative Systems Pharmacology, Early Clinical Development, Pfizer Inc., La Jolla, CA, United States

**Keywords:** anti-PD-1 (programmed cell death-1 protein) monoclonal antibody, anti-PD-L1, immune checkpoint inhibitors, quantitative systems pharmacology (QSP), inter-individual variability, mouse, tumor growth

## Abstract

While anti-PD-1 and anti-PD-L1 [anti-PD-(L)1] monotherapies are effective treatments for many types of cancer, high variability in patient responses is observed in clinical trials. Understanding the sources of response variability can help prospectively identify potential responsive patient populations. Preclinical data may offer insights to this point and, in combination with modeling, may be predictive of sources of variability and their impact on efficacy. Herein, a quantitative systems pharmacology (QSP) model of anti-PD-(L)1 was developed to account for the known pharmacokinetic properties of anti-PD-(L)1 antibodies, their impact on CD8^+^ T cell activation and influx into the tumor microenvironment, and subsequent anti-tumor effects in CT26 tumor syngeneic mouse model. The QSP model was sufficient to describe the variability inherent in the anti-tumor responses post anti-PD-(L)1 treatments. Local sensitivity analysis identified tumor cell proliferation rate, PD-1 expression on CD8^+^ T cells, PD-L1 expression on tumor cells, and the binding affinity of PD-1:PD-L1 as strong influencers of tumor growth. It also suggested that treatment-mediated tumor growth inhibition is sensitive to T cell properties including the CD8^+^ T cell proliferation half-life, CD8^+^ T cell half-life, cytotoxic T-lymphocyte (CTL)-mediated tumor cell killing rate, and maximum rate of CD8^+^ T cell influx into the tumor microenvironment. Each of these parameters alone could not predict anti-PD-(L)1 treatment response but they could shift an individual mouse’s treatment response when perturbed. The presented preclinical QSP modeling framework provides a path to incorporate potential sources of response variability in human translation modeling of anti-PD-(L)1.

## Introduction

Immune checkpoint inhibitors have revolutionized cancer therapy (Robert, 2020). The first monoclonal antibody targeting cytotoxic T-lymphocyte associated protein-4 (CTLA-4) was approved in 2011, followed by the approval of monoclonal antibodies targeting programmed cell death protein 1 (PD-1) and its ligand, PD-L1 (Sun et al., 2018; Stankovic et al., 2019). PD-1 and PD-L1 are two of the most well-studied immunotherapy targets (Dammeijer et al., 2017; Stankovic et al., 2019). PD-1 is expressed on the surface of antigen-stimulated T cells, including both helper, regulatory, and cytotoxic T cells; while PD-L1 is expressed on tumor cells in a wide variety of cancers, including lung cancer, breast cancer, and melanoma (Sun et al., 2018; Zheng et al., 2019). The ligation of PD-1 by PD-L1 inhibits T cell effector functions, such as proliferation, survival, and cytokine production (Sun et al., 2018), in turn, promoting tumor cells to escape immune surveillance. Anti-PD-1 and anti-PD-L1 [hereafter referred to jointly as anti-PD-(L)1] antibodies competitively bind with PD-1 and PD-L1, respectively. Antibody binding to the targets reduces endogenous PD-1 and PD-L1 interaction and allows T cells to resume normal function. Anti-PD-L1 antibodies with an effector function also induce antibody-dependent cellular cytotoxicity (ADCC) of tumor cells (Boyerinas et al., 2015; Kurino et al., 2020). Anti-PD-(L)1 monotherapies have been proven to be effective treatments for many types of cancer, but response rates remain low, with only 18–45% of patients showing complete or partial tumor regression in clinical trials (Sun et al., 2018). Understanding the sources of response variability in the clinic can aid in the prospective identification of patient populations who are more likely to respond to anti-PD-(L)1 treatments, and therefore reduce the number of patients experiencing potentially severe adverse events while receiving no benefit from the treatments (Darvin et al., 2018; Doroshow et al., 2021; Kumar et al., 2021; Wang et al., 2021).

High clinical variability in responses can make data interpretation challenging, especially in Phase 1 trials, where multiple dose levels are investigated using a relatively small number of patients. This hinders the ability to make informed decisions for subsequent trial designs. Rational design, such as patient inclusion and exclusion criteria and dosing regimen/dose selection, is necessary to effectively evaluate PK, safety, and efficacy of therapeutics under investigation. Preclinical data can help predict clinical values, such as PK and efficacious dose levels (Lieu et al., 2013). The data-based predictions are useful in dose selection in clinical trials to achieve full exploration of the expected ranges. Accurate predictions lead to efficient trial design, limiting the cost of clinical trials and providing maximum benefit to trial participants while gathering sufficient information for final dose selection.

In immuno-oncology, preclinical studies frequently use syngeneic mouse models to evaluate therapeutic efficacy due to their intact immune systems, easy preparation, and stable *in vivo* tumor growth (Saito et al., 2020). These models approximate clinical disease as they represent an immune response to acute exposure rather than a tumor that develops over an extended period without necessarily triggering the immune system. Accordingly, translation methods should take these differences in immune response into account when predicting doses for the clinic. Translation methods can vary between simple allometric scaling of PK and doses to more sophisticated methods, such as quantitative systems pharmacology (QSP) modeling (Zhang et al., 2022).

QSP can leverage a greater breadth of preclinical datasets to generate human predictions, which can then be used in designing clinical trials. For example, preclinical QSP models can be used to understand the dynamics of the underlying biological system and response to immune-oncology treatments, and therefore to help identify mechanistic hypotheses for the inter-individual variability observed in tumor volume profiles in syngeneic mouse models following the immuno-oncology treatments. Understanding the variability and uncertainty inherent in preclinical data can streamline translation to first-in-patient studies by allowing the incorporation of variability into clinical model predictions.

We sought to build a methodology for preclinical to clinical translation in situations with high inter-individual variability using anti-PD-(L)1 therapies as an example. Previous models of anti-PD-(L)1 focused on using clinical data to identify potential biomarkers for pembrolizumab and ipilimumab in melanoma (Kumar et al., 2021) and for atezolizumab in breast cancer (Wang et al., 2021). In comparison, a preclinical model leveraged a simpler QSP model to identify potential biomarkers through sensitivity analysis (Valentinuzzi et al., 2019). Herein, we developed a mouse QSP model of anti-PD-(L)1 to investigate potential sources of inter-individual variability. The model leveraged intra-tumoral T cell profiling data to characterize T cell kinetics parameters. It captured variability in the growth of CT26 syngenetic tumors post anti-PD-(L)1 treatments. Further, local sensitivity analysis led to hypotheses of potential biomarkers in mice that can be predictive of response to the treatments. This work lays a foundation for leveraging QSP models for preclinical to clinical translation in situations with high inter-individual variability.

## Methods

### Mouse datasets

All mouse studies were approved by the Pfizer Institutional Animal Care and Use Committee according to established guidelines.

To obtain intra-tumoral T cell profiling data, four immunocompetent Balb/c mice were inoculated with CT26 tumor cells. The tumors were harvested 7 days after inoculation. Frequencies of CD8^+^ inactive T cells [CD8^+^CD4-TCF7+ (Kurtulus et al., 2019)], CD8^+^ active T cells [CD8^+^CD4-PD1+CD44^+^ (Baaten et al., 2010)], CD8^+^ proliferating T cells [CD8^+^CD4-Ki67+ (Scholzen and Gerdes, 2000)], CD8^+^ cytotoxic T-lymphocytes [CTLs, CD8^+^CD4-Granzyme B+ (Pardo et al., 2009)] and CD8^+^ exhausted CTLs [CD8^+^CD4-PD1+Tim3+Lag3+ (Maruhashi et al., 2020; Yang et al., 2020)] out of CD45^+^ T cells were quantified by cell surface markers using flow cytometry.

Tumor growth inhibition data are from five studies of immunocompetent Balb/c mice inoculated with CT26 tumor cells. The mice were treated with phosphate buffer saline (PBS, control), 0.5% methylcellulose (control), water (control), anti-mouse-PD-1 antibody (CD279, RMP1-14, BioXCell®), or anti-mouse PD-L1 antibody (B7-H1, 10F.9G2, BioXCell®). Tumor sizes were measured using calipers. Tumor volumes were calculated as (width x width x length)/2. Every study had a control group and 1-2 anti-PD-(L)1 treatment groups. Details of the five studies are shown in Table 1.

**TABLE 1:** Details of tumor growth inhibition studies with CT26 syngeneic mouse tumor model. Q5D = every 5 days. Q4D = every 4 days. Q3D = every 3 days. QD = every day. IV = intravenously. IP = intraperitoneally. MC = methylcellulose.

Study	Study site ID	Number of mice per group	Control	Anti-PD-1 (RMP1-14)	Anti-PD-L1 (10F.9G2)	Number of tumor cells implanted	Treatment start (days after tumor implantation)	Duration of study (days after tumor implantation)
1	1	10	PBS IV Q5Dx2	10 mg/kg IV Q5Dx2	N/A	2 × 10^6^	8	148
2	1	10	PBS IV Q4Dx4	5 mg/kg IV Q4Dx4	N/A	2 × 10^6^	10	56
3	1	10	PBS IV Q3Dx3	10 mg/kg IV Q3Dx3	10 mg/kg IV Q3Dx3	2 × 10^6^	7	242
4	2	12	0.5% MC oral QD	N/A	10 mg/kg IP Q3Dx3	2 × 10^5^	11	52
5	2	21	Water oral QD	N/A	10 mg/kg IP Q3Dx3	2 × 10^5^	8	40

### Anti-PD-(L)1 QSP model

A QSP model was originally designed to investigate sources of variability in response to anti-CTLA4 (Qiao, 2022) and the model was updated to incorporate the mechanism of action of anti-PD-(L)1. The QSP model consists of three compartments, including the plasma and peripheral compartments to capture the pharmacokinetics of anti-PD-(L)1 antibodies, and the tumor compartment to describe the pharmacodynamics within the tumor microenvironment.

**FIGURE 1:**
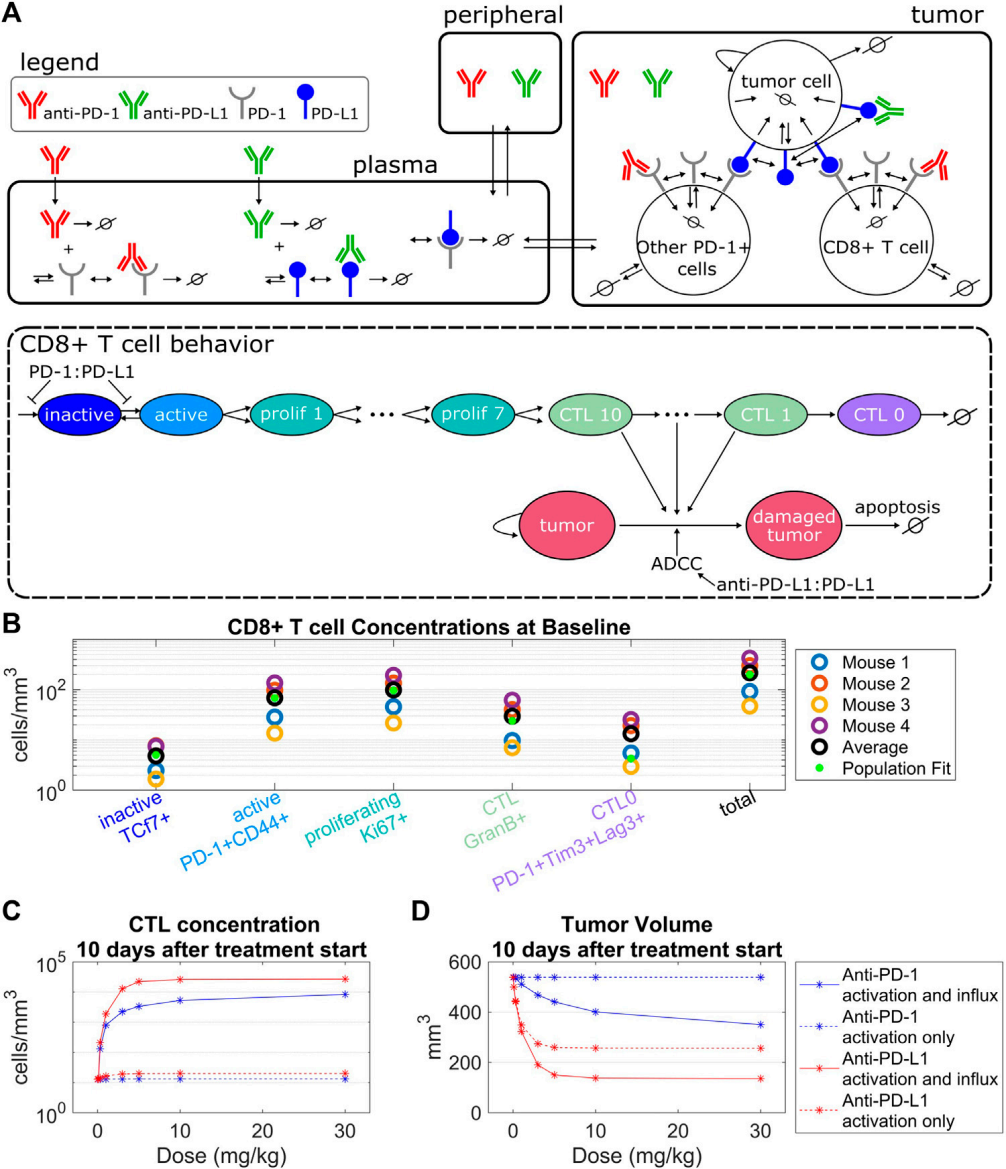
Model structure captures intra-tumoral T cell dynamics from *in vivo* data. **(A)** The QSP model consists of three compartments that describe the pharmacokinetics of anti-PD-(L)1, molecular interactions of the antibodies with their targets, cellular kinetics and interactions of CD8^+^ T cells and tumor cells. The life cycle of the CD8^+^ T cells is depicted in the QSP model. **(B)** CD8^+^ T cell concentrations from flow cytometry data of four mice plotted with the model simulated steady state concentrations using the population fit parameter set. Simulated steady state values of T cell concentrations are depicted by green dots. **(C,D)** Percent changes in CTL concentrations **(C)** and tumor volumes **(D)** from the control simulation 10 days after treatment start for the model with constant CD8^+^ T cell influx vs. the model with PD-1:PD-L1 binding dependent CD8^+^ T cell influx. Treatment of either anti-PD-1 Q3Dx3 or anti-PD-L1 Q3Dx3 is initiated 7 days after tumor implantation.

In the tumor compartment the concentrations of tumor cells, PD-1+CD8^+^ T cells, and other PD-1+ T cells were modeled. Tumor cells are damaged through interactions with CTLs or through anti-PD-L1 ADCC. Damaged tumor cells undergo apoptosis at a constant rate. Other PD-1+ T cells serve as an alternate binding site for PD-L1 on tumor cells. PD-1+CD8^+^ T cells include CD8^+^ T cells in the inactive, active, proliferating, CTL, and exhausted CTL stages depicted in Figure 1A. PD-1+CD8^+^ T cells are synthesized as inactive cells ( $E^{I}$ ). The cells go through eight proliferation stages ( $E^{A}$ , and $E^{P_{i}}$ for i=1..7 ) before becoming CTLs ( $E^{D_{i}}$ for i=1...10 ) (Qiao, 2022). A total of eight proliferation stages was chosen to match the magnitude of T cell was chosen to match the magnitude of T cell expansion observed in (Yoon et al., 2010). Each CTL can damage ten tumor cells before becoming exhausted ( $E^{D_{0}}$ ) (Halle et al., 2016). All CTLs are assumed to be reactive towards the tumor. All PD-1+CD8^+^ T cells express the same number of PD-1 receptors per cell. Inactive, active, and proliferating CD8^+^ T cells degrade at a rate of $k_{d,CD8}$ while CTLs degrade at a rate of $k_{d,CTL}$ .

The binding of PD-L1 expressed on tumor cells to PD-1 expressed on inactive PD-1+CD8^+^ T cells ( $P^{I}:L^{V}$ ) controls T cell behavior. This binding inhibits the influx ( $\left.f_{influx}\right)$ of inactive PD-1+CD8^+^ T cells into the tumor microenvironment, and the activation ( $f_{activate}$ ) of PD-1+CD8^+^ T cells in the tumor compartment (Eqs 1–3). In the absence of antibody treatments, PD-1:PD-L1 per inactive CD8^+^ T cell is at its steady state concentration (approximately 6.6 PD-1:PD-L1 per inactive CD8^+^ T cell) causing inactive PD-1+CD8^+^ T cells to enter the tumor compartment at the rate of $k_{s,I}V_{t}$ (Eq. 2). The influx rate ensures that the CD8^+^ T cells reach a steady state concentration during control simulations. The maximum influx and activation rates are denoted $E_{\max}^{influx}$ and $E_{\max}^{activate}$ , respectively. Both functions reach half maximal values at EC50 .

$$\frac{dE^{I}}{dt}=f_{influx}\left(\frac{P^{I}:L^{V}}{E^{I}}\right)-k_{d,CD8}E^{I}-f_{activate}\left(\frac{P^{I}:L^{V}}{E^{I}}\right)E^{I}+k_{A2Ibasal}E^{A}$$ (1)

$$f_{influx}\left(\frac{P^{I}:L^{V}}{E^{I}}\right)=\max\left[0,\frac{E_{\max}^{influx}}{1+\frac{EC50}{6.6}}-\frac{E_{\max}^{influx}}{1+\frac{EC50}{\frac{P^{I}:L^{V}}{E^{I}}}}\right]+k_{s,I}V_{t}$$ (2)

$$f_{activate}\left(\frac{P^{I}:L^{V}}{E^{I}}\right)=E_{\max}^{activate}-\frac{E_{\max}^{activate}}{1+\frac{EC50}{\frac{P^{I}:L^{V}}{E^{I}}}}$$ (3)

Equations describing the subsequent T cell stages follow the set up in (Qiao, 2022) and can be found with the full model equations in Supplementary Material.

### Model fitting

Parameters describing receptor expression, antibody half-lives, binding constants, and plasma concentrations were sourced from relevant literature (Supplementary Table S1). The unknown model parameters were estimated in three stages. First, pharmacokinetic parameters of RMP1-14 (anti-PD-1) and 10F.9G2 (anti-PD-L1) were fit from the published single dose data (Zalba et al., 2020) using two-compartment models with linear elimination (Supplementary Figure S1).

Second, T cell kinetic parameters including maximum CD8^+^ T cell activation rate, T cell proliferation half-life, CD8^+^ T cell half-life, CTL half-life, CTL-mediated tumor cell killing rate, and baseline influx rate of inactive CD8^+^ T cells were constrained using average concentrations of CD8^+^ T cells from untreated CT26 tumors in Balb/c mice that were derived from flow cytometry data. Full details can be found in Supplementary Material.

Third, the full QSP model was fit to tumor volume profiles from five *in vivo* mouse studies. Non-linear mixed effects (NLME) model fitting and local sensitivity analysis were performed iteratively to identify the key parameters to capture inter-individual variability. Local sensitivity analysis was centered on the population parameter estimates. In each iteration, sensitive parameters were included as random effects in the NLME model. Based on the results of fitting the inter-individual variability, study site ID of the five *in vivo* mouse studies was identified as a significant covariate. An exponential covariate model was included to capture study site level effects. Goodness of fit was evaluated using Akaike information criteria, Bayesian information criteria and visual inspection of individual fits. The control and treatment groups of all the studies were fit simultaneously.

Model fitting was performed in Monolix 2020R1 (Lixoft SAS, a Simulations Plus company). Local sensitivity analysis was performed in MATLAB version R2020b (MathWorks software). Plots were generated in R version 4.0.3.

### Local sensitivity analysis

Local sensitivity analysis was performed using either the population fit or a chosen fitted animal as the starting parameter set. Parameters were perturbed ± 30% one at a time. The percent change in the area under the tumor volume curve (AUC) was calculated for control, anti-PD-1, and anti-PD-L1 treatment simulations. Three doses of 10 mg/kg given every 3 days (Q3D) were simulated with the first dose administered on day 7. The AUC was chosen to summarize the changes to tumor volume over the entire 30 days.

Local sensitivity analysis was performed in MATLAB version R2020b (MathWorks software). Plots were generated in R version 4.0.3.

## Results

### T cell influx as a function of PD-1 to PD-L1 binding is necessary to model antibody-induced anti-tumor response while capturing intra-tumoral T cell dynamics

The QSP model describes the pharmacokinetics of anti-PD-(L)1, interactions between the antibodies and their molecular targets, tumor cell dynamics, intra-tumoral T cell dynamics, and CTL-mediated tumor cell killing (Figure 1A). Five stages of T cell development were tracked in the tumor compartment, described as inactive, active, proliferating, cytotoxic, and exhausted T cells. The concentration of each of these T cell populations depends on the T cell kinetic parameters including baseline CD8^+^ T cell influx rate, CD8^+^ T cell half-life, CTL half-life, maximum CD8^+^ T cell activation rate, CD8^+^ T cell proliferation half-life, and CTL-mediated tumor cell killing rate. These parameters were constrained using the average concentrations of CD8^+^ T cells from untreated CT26 tumors in Balb/c mice (Figure 1B, Supplementary Table S1) that were derived from flow cytometry data where the five CD8^+^ T cell populations were defined by cell surface markers (Supplementary Material).

The maximum CD8^+^ T cell activation rate limits the maximum amount of change in the CTL concentration post antibody treatments. Specifically, simulations indicate that anti-PD-1 treatments administered Q3Dx3 induced a maximum increase of 2.8% in CTL concentration and 0.017% reduction in tumor volume 10 days after treatment initiation compared to control simulations, whereas anti-PD-L1 treatment administered Q3Dx3 induced a maximum increase of 56% in CTL concentration and 52% reduction in tumor volume (Figures 1C,D). Differences in response to anti-PD-1 and anti-PD-L1 treatments is due to differences in receptor occupancy and the ADCC effect of anti-PD-L1. These simulations indicated that activation and expansion of resident CD8^+^ T cells in the tumor microenvironment alone are insufficient for modeling the anti-tumor responses of anti-PD-(L)1 treatments.

When a PD-1:PD-L1 interaction dependent CD8^+^ T cell influx was incorporated, the increase in CTL concentration due to anti-PD-(L)1 treatments significantly increased (from 2.8% to 65000% for anti-PD-1, and from 56% to 208000% for anti-PD-L1), as did the reduction in tumor volume (from 0.017% to 35% for anti-PD-1, and from 52% to 75% for anti-PD-L1) (Figures 1C,D).

In summary, intratumor T cell profiling data were used to characterize kinetic parameters of CD8^+^ T cells in CT26 + tumor microenvironment. Model simulations indicate that dependence of the influx of CD8^+^ T cells to the tumor microenvironment on the amount of PD-1:PD-L1 interactions between T cells and tumor cells is necessary for anti-PD-(L)1-mediated anti-tumor responses.

### Tumor volume profiles reveal inter-individual and inter-study variabilities in addition to dose-dependent responses to anti-PD-1 treatment

**FIGURE 2:**
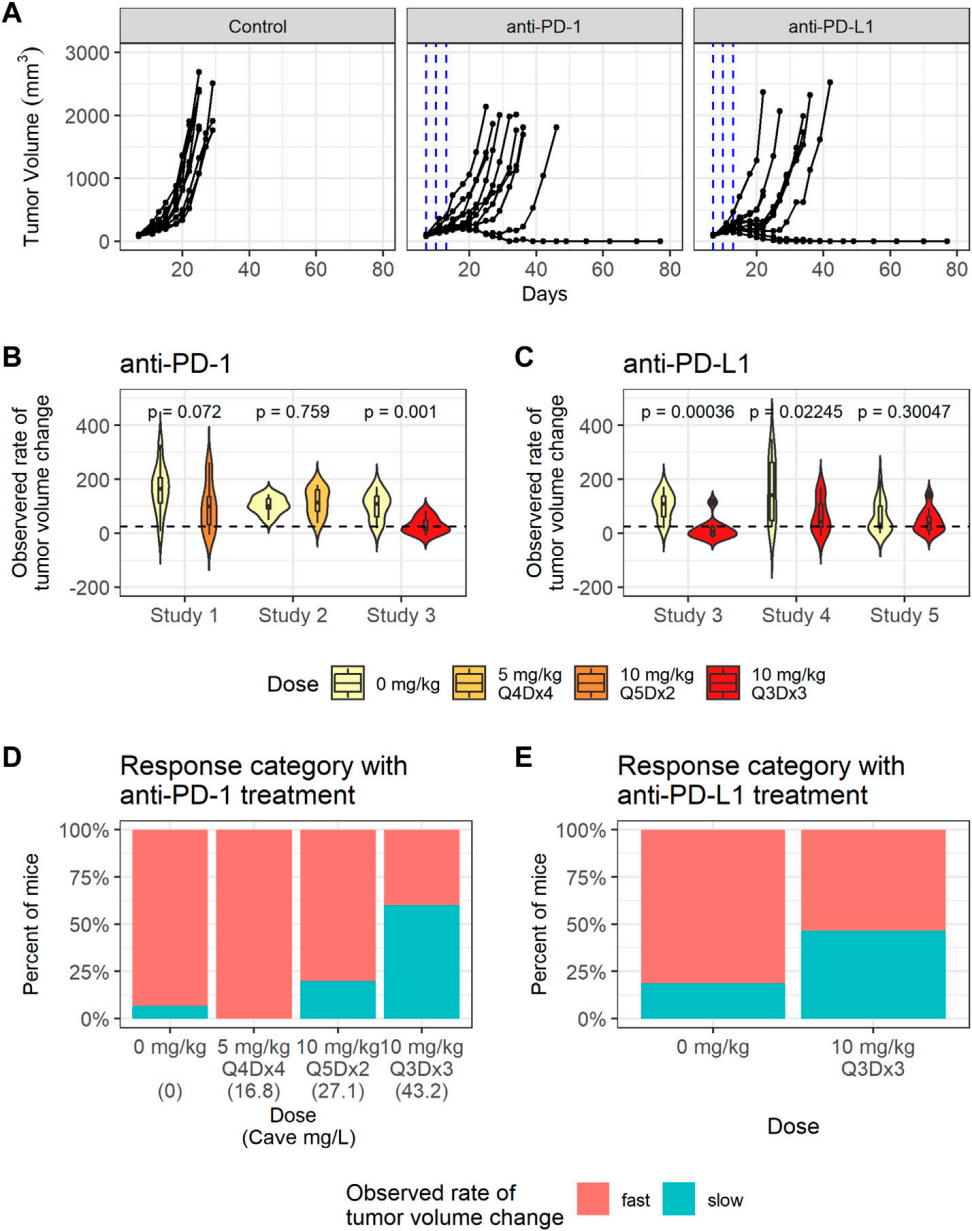
CT26 tumor growth inhibition data exhibit dose dependent response to anti-PD-1. **(A)** Representative individual tumor volume profiles of the control group, and the individual tumor volume profiles after treatment with anti-PD-1 administered at 10 mg/kg Q3Dx3 or anti-PD-L1 administered at 10 mg/kg Q3Dx3. **(B)** Violin plots showing observed rates of tumor volume changes between day 7 and day 13 post treatments with PBS (control) or anti-PD-1 in studies 1–3. Dashed line indicates the threshold (25 mm^3^/day) for classifying slow- and fast-growing tumors. **(C)** Violin plots showing observed rate of tumor volume changes during between day 7 and day 13 post treatments with PBS (control) or anti-PD-L1 in studies 3–5. **(D)** Percent of control and anti-PD-1 treated mice with slow-growing tumors between day 7 and day 13 after treatment start in studies 1–3 sorted according to simulated average plasma anti-PD-1 concentration (Cave). **(E)** Percent of control and anti-PD-L1 treated mice with slow-growing tumors between day 7 and day 13 after treatment start in studies 3–5.

Significant variability in tumor volume profiles and responses to anti-PD-(L)1 treatments was observed in the *in vivo* studies with the CT26 syngeneic mouse tumor model. The dataset consisted of 136 mice with CT26 tumors treated with PBS, 0.5% MC or water (control group), anti-PD-1 antibody (RMP1-14) or anti-PD-L1 antibody (10F.9G2) (treatment details in Table 1). Representative data are shown in Figure 2A and the full dataset is shown in Supplementary Figure S4.

To quantify the differences in individual tumor volume profiles, the observed rates of tumor volume change between day 7 and day 13 after treatment start were calculated for each mouse. The observed rates of tumor volume change varied significantly between studies, as identified through a Kruskal-Wallis rank sum test (*p* = 0.0054).

The average observed rates of tumor volume change between the treatment group and the control group within each study were tested for significance using a Wilcoxon rank sum test. As shown in Figures 2B,C, anti-PD-1 treatment caused a statistically significant decrease in the observed rates of tumor volume change only at the highest dose (study 3, 10 mg/kg Q3Dx3). Anti-PD-L1 treatment caused statistically significant decreases in the observed rates of tumor volume change in studies 3 and 4, but not in study 5 (*p*-values are unadjusted, significance threshold is 0.01/6 = 0.0016 with Bonferroni correction) despite the same dosing regimen being used in the studies and similar tumor sizes at treatment start in studies 3 and 5 (Supplementary Figure S2, Supplementary Table S2).

Tumors with observed rates of change below 25 mm^3^ per day were classified as having a slow rate of change, while the others were classified as having a fast rate of change. The threshold of 25 mm^3^ per day was chosen as it identifies all mice that experience tumor regression after treatment, or a period of regression followed by progression (Supplementary Figure S3). While mice with fast tumor growth are present in all the dose groups, the number of tumors with a slow observed rate of tumor volume change increased as the anti-PD-1 dose increased (Figure 2D), indicating dose response.

In summary, substantial inter-study and inter-individual variability is present in the tumor volume profiles. Anti-PD-1 led to dose response in tumor growth inhibition, whereas dose ranging data were not available for anti-PD-L1 (Figure 2E).

### The QSP model explains sources of variability in baseline growth and treatment response observed in tumor volume profiles post anti-PD-(L)1 treatments

**FIGURE 3:**
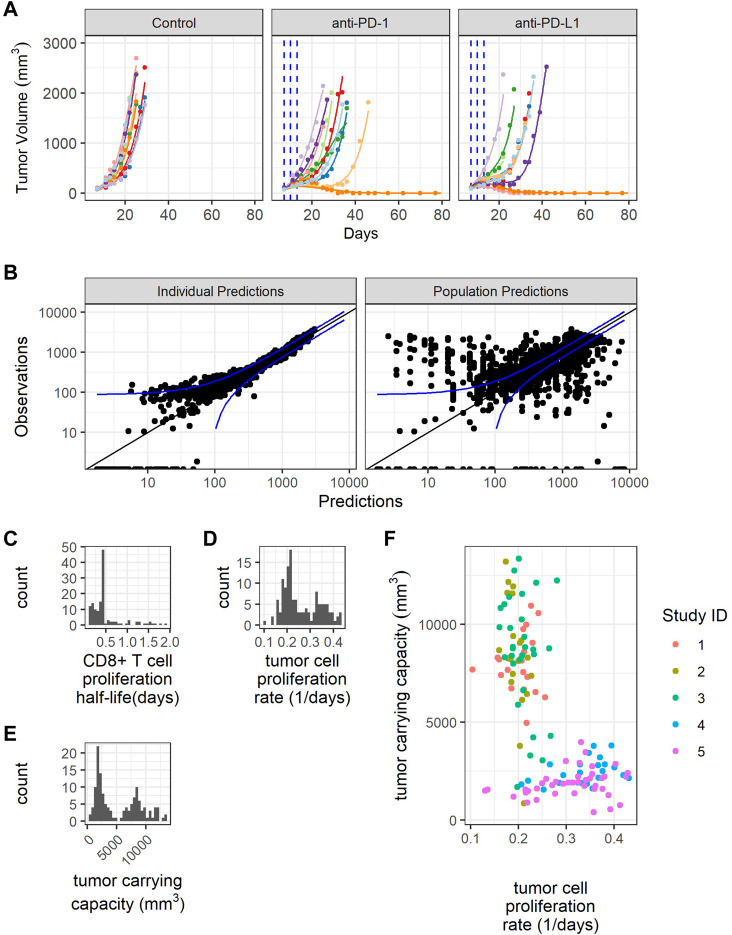
Model fitting recapitulates inter-individual variability. **(A)** Representative comparison (study 3) between observed tumor volume profiles (colored dots) and individual simulations (colored lines) for study 3. **(B)** Observations verses predictions. **(C)** Distribution of model estimated individual PD-1+CD8^+^ T cell proliferation half-lives. **(D)** Distribution of model estimated individual tumor cell proliferation rates. **(E)** Distribution of model estimated individual tumor carrying capacities. **(F)** Individual tumor cell proliferation rates and tumor carrying capacities show study dependent distributions.

Next, we parameterized the QSP model to understand the variabilities observed in the tumor volume profiles post anti-PD-(L)1 treatments. PK parameters for RMP1-14 and 10F.9G2 were estimated based on literature data (Zalba et al., 2020) (Supplementary Figure S1). The tumor volume-time profiles were used to fit the CD8^+^ T cell proliferation half-life, maximum rate of ADCC, tumor cell apoptosis rate, maximum rate of CD8^+^ T cell influx, CTL-mediated tumor cell killing rate, tumor cell proliferation rate, and tumor carrying capacity (Supplementary Table S1). Model fitting was approached iteratively, alternating between local sensitivity analysis and NLME modeling to identify a minimum number of parameters to describe the inter-study and inter-individual variabilities in the tumor volume profiles. The final model included random effects for CD8^+^ T cell proliferation half-life, tumor cell proliferation rate and tumor carrying capacity, and included study site ID as a covariate for population tumor cell proliferation rate and tumor carrying capacity (Table 2). The model was sufficient to replicate the variation in the tumor volume profiles (Figure 3A, Supplementary Figure S4, and Table 2). Removing the random effects for any of the three parameters diminished the goodness of fit (results not shown).

**TABLE 2:** Fitted model parameters. Parameter symbols used in the equations are shown in parentheses.

Parameter	Description	Unit	Value	R.S.E. (%)	Shrinkage (%)
**Fixed effect**					
ThalfCD8pfr_pop	CD8^+^ T cell proliferation half-life	Days	0.418	44.9	−
ADCC_Emax_pop $\left(E_{\max}^{ADCC}\right)$	Max ADCC rate	1/day	0.146	5.63	−
Kapop_TC_M_pop $\left(k_{apop}\right)$	Damaged tumor cell apoptosis rate	1/day	0.191	11.2	−
Kpfr_TC_pop $\left(k_{pfr,T}\;\right)$	Population tumor cell proliferation rate	1/day	0.199	2.68	−
Beta_kpfr_TC_STUDY_2	Effect of study site ID on tumor cell proliferation rate		0.444	8.54	−
Klimit_TC_pop $\left(k_{limit,T}\right)$	Population tumor carrying capacity	mm^3^	8,370	14.5	−
Beta_klimit_TC_STUDY_2	Effect of study site ID on tumor carrying capacity		−1.47	11.5	−
Influx_Emax_pop $\left(E_{\max}^{influx}\right)$	Maximum influx of CD8^+^ inactive T cells	Cells/day	8.43×10^7^	1.75	−
**Random effect**					
Omega_ThalfCD8pfr	Standard deviation of the random effect on the CD8^+^ T cell proliferation half-life	Days	3.75	34.7	88.1
Omega_kpfr_TC	Standard deviation of the random effect on the tumor cell proliferation rate	1/day	0.21	6.51	6.97
Omega_klimit_TC	Standard deviation of the random effect on the tumor carrying capacity	mm^3^	3.67×10^3^	11.0	49.6
**Error model (combined 2)**					
a	Constant term		53.0	3.56	
b	Proportional term		0.142	4.41	

The individual predictions recapitulate the observations, except for very small tumor sizes (Figure 3B). The distribution of CD8^+^ T cell proliferation half-life is clustered around the average value, indicating difficulty estimating the distribution given the available data (Figure 3C). The distributions of individual tumor cell proliferation rates and tumor carrying capacities show a bimodal distribution (Figures 3D,E). The tumors in studies 1-3 tend to have lower tumor cell proliferation rates and higher carrying capacities, while the tumors in studies 4 and 5 have higher tumor cell proliferation rates and lower carrying capacities (Figure 3F).

In summary, the model replicates the variability in CT26 tumor volume-time profiles by incorporating inter-individual variations in three parameters, namely, CD8^+^ T cell proliferation half-life, tumor cell proliferation rate, and tumor carrying capacity.

### Additional parameters change treatment response when perturbed

We utilized the individual parameter estimates to identify the sources of variability in anti-tumor responses. Anti-tumor responses to anti-PD-(L)1 treatments were still categorized based on the observed rates of tumor volume change between day 7 and day 13 after treatment initiation. Mice with an observed rate of tumor volume change below 25 mm^3^/day were considered responders, while all others were considered non-responders.

**FIGURE 4:**
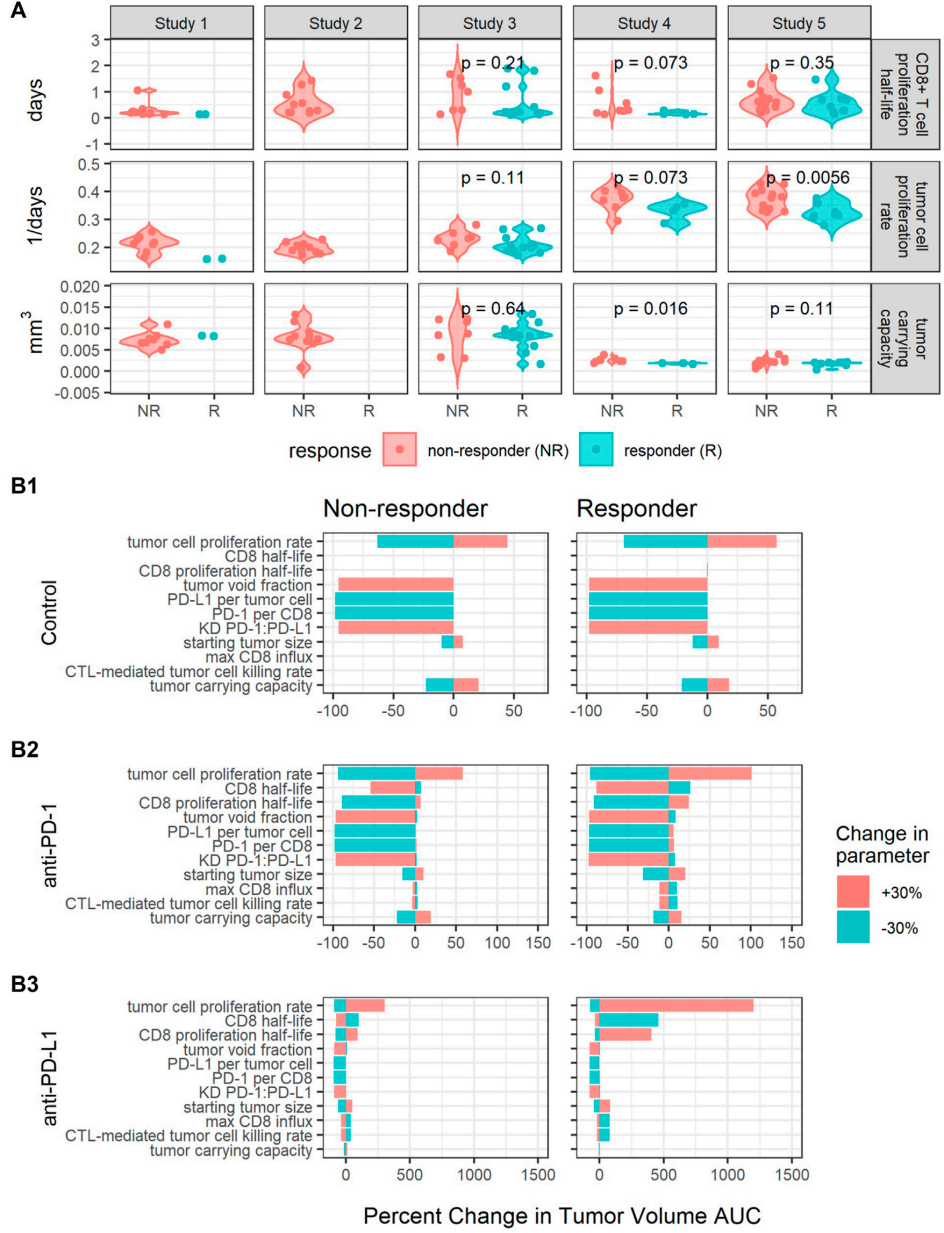
Perturbations in key parameters change individual mice’s responses. **(A)** Comparisons of CD8^+^ T cell proliferation half-lives, tumor cell proliferation rates, and tumor carrying capacities between responders (R) and non-responders (NR) for each study. *p*-values were calculated using the Wilcoxon rank sum test. **(B)** Local sensitivity analysis centered on parameters sets fit to a responder and a non-responder from Study 5 for control **(B1)**, anti-PD-1 **(B2)** and anti-PD-L1 **(B3)** simulations. Model output is summarized as the percent changes in the area under the tumor volume curve (AUC) over 30 days. The parameters are listed in the order of decreasing sensitivity across all simulations.

Estimated values for individual parameters including CD8^+^ T cell proliferation half-life, tumor cell proliferation rate, and tumor carrying capacity were compared between responders and non-responders (Figure 4A). The CD8^+^ T cell proliferation half-lives and tumor carrying capacities do not significantly differ between responders and non-responders across studies (*p*-values are unadjusted, significance threshold is 0.05/3 = 0.016 with Bonferroni correction), potentially due to the high shrinkage values (Table 2). The average tumor cell proliferation rates significantly differ between responders and non-responders in study 5, and the tumor cell proliferation rate correlates with the observed rate of tumor volume change between day 7 and day 13 after treatment start for most studies and treatments (Supplementary Figure S5). The study-specific findings remain exploratory due to small sample sizes.

Furthermore, we sought to identify additional parameters that affect treatment response through local sensitivity analysis centered on parameter sets fitted to two mice with different responses to anti-PD-L1 treatment. The two mice were selected from the same study to have similar tumor cell proliferation rates and tumor carrying capacities and to respond to anti-PD-L1 10 mg/kg Q3Dx3 treatment differently. In simulations, the responder shows complete tumor regression in response to both 10 mg/kg Q3Dx3 anti-PD-1 and 10 mg/kg Q3Dx3 anti-PD-L1, while the non-responder shows continued tumor growth when treated with either anti-PD-1 or anti-PD-L1 (data not shown). For the local sensitivity analysis, each parameter was perturbed by 30% in both directions from the nominal parameter value one at a time. The new parameter set was used to simulate tumor volume-time profiles under the same conditions. The simulated AUCs were compared to the simulated AUCs with the nominal parameter values (Figure 4B).

In the control simulations, increasing the PD-1:PD-L1 dissociation constant (KD) or the fraction of tumor volume not occupied by cells (tumor void fraction) caused the tumor to completely regress. Decreasing the tumor cell proliferation rate, PD-L1 per tumor cell, or PD-1 per CD8^+^ T cell also caused the tumor to regress. The results suggest these intrinsic tumor properties determine the growth of the tumor in the absence of treatments (Figure 4B1).

The intrinsic tumor cell proliferation rate is also an important determinant of treatment response. A 30% decrease in the tumor cell proliferation rate caused the non-responder to experience complete tumor regression in both anti-PD-1 and anti-PD-L1 treatment simulations. Similarly, a 30% increase in the tumor cell proliferation rate caused the responder to experience a large increase in tumor volume (Figures 4B2,B3). This is consistent with the difference in average tumor cell proliferation rate between responders and non-responders identified in Figure 4A.

Increasing PD-L1 expression has a minimal impact on treatment response due to high receptor engagement during either treatment. At the nominal PD-L1 expression level, during anti-PD-1 simulations, receptor engagement can drop from approximately 85% to as low as 53% between doses. Consequently, increasing PD-L1 expression induced a 0.9% and 6.1% increase in tumor volume AUC for the non-responder and responder, respectively (Figure 4B2). In anti-PD-L1 simulations, increasing PD-L1 had minimal impact on AUC (Figure 4B3) due to PD-L1 receptor occupancy being maintained at >97% during treatment. For both treatments, 10 mg/kg Q3Dx3 is near the top of the dose response curve (Figures 1C,D), and a small increase in receptor expression is insufficient for anti-PD-(L)1 treatments to lose efficacy.

Properties of the immune system such as CD8 half-life, CD8 proliferation half-life, max CD8^+^ T cell influx rate, and the CTL-mediated tumor cell killing rate influenced response to anti-PD-(L)1 treatments (Figures 4B2,B3) but had no impact on tumor growth during control simulations (Figure 4B1). Decreasing the CD8^+^ T cell proliferation half-life causes the non-responder AUC to decrease 89% and 79% during anti-PD-1 and anti-PD-L1 treatments respectively while the opposite change causes the responder to experience a 24% and 405% increase in AUC, respectively. Similarly, in the non-responder, increasing the CD8^+^ T cell half-life causes a 54% and 75% decrease in AUC during anti-PD-1 and anti-PD-L1 treatments respectively while for the responder, decreasing the CD8^+^ T cell half-life causes a 26% and 460% increase in AUC. Smaller percent changes are seen in the responses to perturbations in the max CD8^+^ T cell influx rate and the CTL-mediated tumor cell killing rate.

In summary, while random effects on tumor carrying capacity, tumor cell proliferation rate, and CD8^+^ T cell proliferation half-life explain the variability in the profiles of CT26 tumors post anti-PD-(L)1 treatments, baseline tumor growth and treatment response are sensitive to both intrinsic tumor properties and immune system properties. Variability beyond the three parameters fit to individuals in this analysis should be considered to improve the prediction intervals in future analyses.

### Treatment response is dependent on PD-1 and PD-L1 expression levels but not independent of the tumor cell proliferation rate

**FIGURE 5:**
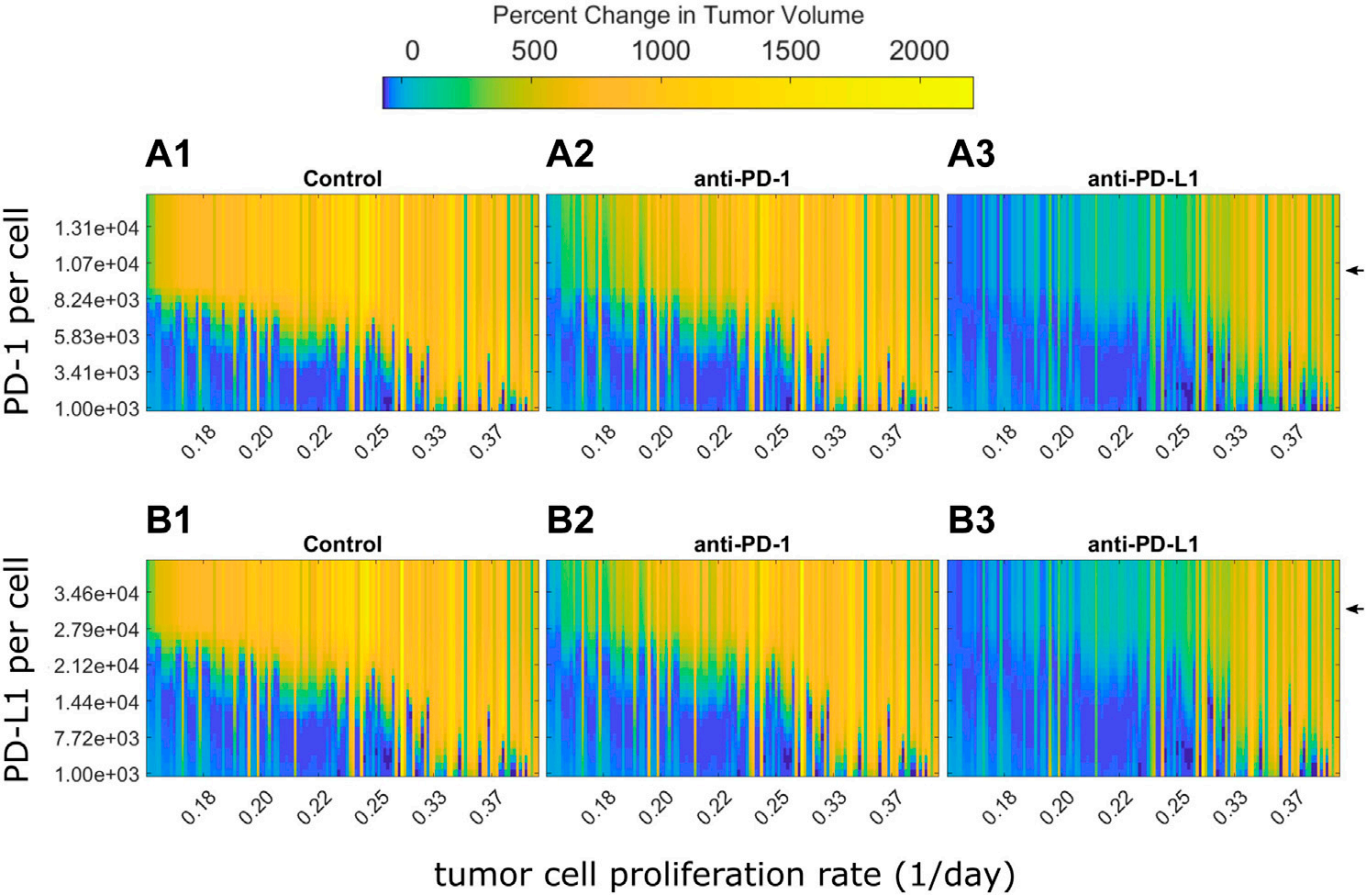
Optimal PD-1 or PD-L1 expression level for treatment response differs between individuals. Percent changes in tumor volume 12 days after treatment start for each fitted mouse with varying PD-1 or PD-L1 expression levels. Mice are arranged on the x-axis according to their estimated tumor cell proliferation rates. PD-1 and PD-L1 expression levels used in QSP model fitting is marked by black arrows. **(A)** Change in tumor size from treatment start to 12 days after treatment start for fitted mice with varying PD-1 per CD8^+^ T cell for **(A1)** control, **(A2)** 10 mg/kg anti-PD-1 Q3D x3, and **(A3)** 10 mg/kg anti-PD-L1 Q3D x3 simulations. **(B)** Change in tumor size from treatment start to 12 days after treatment start for QSP model fitted mice with varying PD-L1 per tumor cell for **(B1)** control, **(B2)** 10 mg/kg anti-PD-1 Q3D x3, and **(B3)** 10 mg/kg anti-PD-L1 Q3D x3 simulations.

PD-1 and PD-L1 expression levels are potential sources of variability in clinical populations. In analysis of clinical trial results, patients are often stratified by PD-L1 expression (Doroshow et al., 2021). The preclinical model used here assumes all mice had the same PD-(L)1 expression levels. However, the preclinical QSP model can be used to examine how the modeled tumor volume for each mouse changes in response to different PD-1 and PD-L1 expression levels. For each fitted animal parameter set, the PD-1 or PD-L1 expression level was varied over a range of values and the model was used to predict tumor growth under the control, anti-PD-1, or anti-PD-L1 treatment. Figure 5 shows the percent changes in tumor volume from treatment start to 12 days after treatment start. Mice are ordered along the x-axis by estimated tumor cell proliferation rates. Day 12 was chosen as it is late enough to see differences between responders and non-responders, yet early enough to show differences in the strength of response. Experimentally quantified expression levels are marked by black arrows. Results stratified by study can be found in Supplementary Figures S6, S7.

The model simulations indicate that low PD-(L)1 expression was associated with spontaneous tumor regression in mice with low estimated tumor cell proliferation rates (Figures 5A,B). Tumor volume increases as PD-(L)1 expression levels or estimated tumor cell proliferation rates increase. Anti-PD-(L)1 treatments suppress tumor growth for higher expression levels compared to the control group. Mice with high estimated tumor cell proliferation rates show treatment resistance at all PD-(L)1 expression levels. Mice with a tumor cell proliferation rate below 0.33 per day show an inverted bell shape response curve as receptor expression increases, shown in the transition from light blue to dark blue and returning to light blue as receptor expression increases in each column. This indicates that each tumor cell proliferation rate has a different range of receptor expression levels where the treatment has the highest effect suggesting that dosing levels could be adjusted to each individual for the maximum effect.

In summary, PD-1 and PD-L1 expression levels determine treatment response, but not independent of the tumor cell proliferation rate. Mice with higher receptor expression levels or faster growing tumors may benefit from higher dose levels to saturate the receptors.

## Discussion

Quantitative systems pharmacology models can be used to inform preclinical to clinical translation and dose selection for first-in-patient studies. Often the models are characterized based on pooled preclinical data and translated to generate predictions for an average human patient. Immune checkpoint inhibitors are promising treatments for many forms of cancers; however, patients’ responses are highly variable. When either preclinical or clinical treatment responses are heterogenous, models built on aggregated data fail to represent the full spectrum of responses observed in clinical studies. Herein, we developed a QSP model of anti-PD-(L)1 treatments in Balb/c mice bearing CT26 syngeneic tumors to identify sources of variability in preclinical anti-tumor response, and generated hypotheses for potential sources of variability for pre-clinical to clinical translation.

The QSP model incorporates knowledge from literature including the mechanism of action of anti-PD-(L)1 antibodies, the biology of their molecular and cellular targets, and their effect on T cell activation. The model summarizes the progression of PD-1+CD8^+^ T cells in five stages, namely inactive, active, proliferating, cytotoxic, and exhausted. Intra-tumoral T cell concentration data were used to characterize the rate of progression through these stages at baseline, which determines the magnitude of increase in the CTL concentration upon anti-PD-(L)1 treatments. The inhibitory effect of PD-1:PD-L1 binding between CD8^+^ T cells and tumor cells on the influx of inactive CD8^+^ T cells into the tumor microenvironment is a novel mechanism in the presented model. The association between PD1:PD-L1 binding and infiltration of inactive CD8^+^ T cells is necessary for predicting anti-PD-(L)1 treatment responses while maintaining the observed baseline CD8^+^ T cell distribution across the five stages. The influx mechanism represents activation of naïve T cells within the lymph nodes which then travel to the tumor microenvironment and is supported by recent publications (Dangaj et al., 2019; Yost et al., 2019; Dammeijer et al., 2020; Wu et al., 2020; Dolina et al., 2021). Blocking PD-1 or PD-L1 receptors on inactive CD8^+^ T cells or tumor cells, respectively, increases the influx and activation of CD8^+^ T cells in the tumor microenvironment that ultimately differentiate into the CTL population for tumor cell killing.

The use of intra-tumoral T cell concentration data was necessary to understand the baseline tumor microenvironment, increase confidence in baseline model behavior, reduce parameter uncertainty, and justify the new model mechanism of PD-1:PD-L1 dependent T cell influx. Ideally, longitudinal data showing intra-tumoral T cell concentrations before and after treatments would support fitting this model mechanism; however, generating longitudinal data of this nature is expensive and time expensive and time consuming. Nevertheless, this type of data is necessary for building mechanistic building mechanistic understanding of the biology and pharmacology and deriving meaningful results and well-supported hypotheses from QSP modeling. Further, QSP modeling allows incorporation and translation of preclinical data to inform and augment clinical modeling efforts.

In the clinic, treatments and tumor measurements are continued until disease progression or censoring. Treatment response is categorized using Response Evaluation Criteria in Solid Tumor (RECIST) criteria (Eisenhauer et al., 2009) and the most comparable model output is tumor diameter at each time point. However, in the preclinical *in vivo* studies, mice were given a defined number of doses and tumor volumes were collected after treatment cessation until study termination. As a result, disease progression due to treatment cessation or acquired resistance is indistinguishable in the preclinical *in vivo* data. Grouping treatment response by the rate of tumor volume change between day 7 and day 13 after treatment start allows for identification of mice whose tumor progression is slowed as well as reversed.

Variability in the tumor volume profiles were explained with three random effects, namely, CD8^+^ T cell proliferation half-life, tumor cell proliferation rate, and tumor carrying capacity. Parameter estimates were similar to the previous anti-CTLA4 model (Valentinuzzi et al., 2019). Random effects on the tumor cell proliferation rate and tumor carrying capacity were both necessary to replicate the inter-individual and inter-study variability in the control groups. There was difficulty fitting individual estimates of CD8^+^ T cell proliferation half-life (Table 2), but inclusion of the random effect was necessary to replicate the variability in treatment response. Future datasets defining intra-tumoral T cell concentrations post treatments would increase confidence in these parameter estimates and help to characterize qualities of responders and non-responders. Currently the model does not take inter-individual variability due to PK into account, due to the lack of data and greater sensitivity to other parameters. Incorporating individual PK datasets or published population PK models could increase confidence in model fits by reducing the unexplained variability in response.

The three random effects have been identified as quantities of interest in determining response to various therapies. The tumor cell proliferation rate can be quantified through tumor doubling time which is a prognostic for survival and disease progression in various cancers (Tubiana and Courdi, 1989; Kay et al., 2019). The tumor carrying capacity represents various factors of the tumor microenvironment, such as tissue vascularization and presence of metabolic waste products (Aherne et al., 2020). Other publications have investigated matching tumor growth curves using time variant carrying capacity (Hahnfeldt et al., 1999), or modeling the effect of radiation therapy as a decrease in the carrying capacity (Zahid et al., 2021). Lastly, individuals with metastatic melanoma with a shorter tumor infiltrating lymphocyte doubling time had a higher response frequency to treatment with autologous tumor-infiltrating lymphocytes and interleukin 2 (Aebersold et al., 1991; Rosenberg et al., 1994). The PD-L1 expression level per tumor cell was taken from *in vitro* data of INF-γ treated tumor cells. INF-γ upregulates PD-L1 expression suggesting the value used in the presented model may represent the high end of the realistic range (Abiko et al., 2015; Mimura et al., 2018; Qian et al., 2018). While an imperfect biomarker, PD-L1 expression is the only approved biomarker for selection of PD-L1 positive non-small cell lung cancer patients for anti-PD-(L)1 therapies (Gridelli et al., 2017). Model results suggest that too high of PD-L1 expression inhibits treatment response, but this can be countered with higher dose levels.

The density of tumor infiltrating lymphocytes have been explored as a biomarker for anti-PD-(L)1 treatment response (Yang et al., 2018; Sato et al., 2021). However, the baseline total CD8^+^ T cell concentration was not identified as a sensitive parameter during the local sensitivity analysis. Instead, the model of anti-tumor response is more sensitive to parameters that influence the CTL concentration after anti-PD-(L)1 treatments, such as the CD8^+^ T cell proliferation half-life or maximum CD8^+^ T cell influx rate. Variability in T cell influx after treatments may be caused by variability in macrophage subtypes (Qu et al., 2020).

Influx of T cells into the tumor comes from the peripheral blood or the lymph nodes. Other published models of anti-PD-(L)1 have included the lymph nodes (Kosinsky et al., 2018; Wang et al., 2019). While reaction and diffusion rates were supported by literature, T cell quantities within the lymph nodes remain unvalidated. T cell profiling data of the lymph nodes would provide support for quantitative modeling efforts and increase their predictive power. Herein, the lymph nodes were not explicitly modeled and instead implicitly included in the tumor compartment. This simplification is acceptable for the scope of this modeling effort to allow for investigation into the main sources of variability without including unverifiable quantities.

The presented model structure enables investigation into potential sources of preclinical variability in anti-tumor response as well as potential sources of variability in clinical responses. Varying the tumor cell proliferation rate, tumor carrying capacity, and CD8^+^ T cell proliferation half-life together was enough to capture the inter-study and inter-individual variability in the preclinical *in vivo* data. The tumor cell proliferation rate strongly correlates with the observed rate of tumor volume change after treatments, but the tumor cell proliferation rate alone is unable to determine whether a tumor will respond to anti-PD-(L)1 treatments. Other parameters, such as the CD8^+^ T cell half-life and maximum CD8^+^ T cell influx rate may be additional sources of variability in response to anti-PD-(L)1 treatments as they impact the increase in CD8^+^ T cells after treatments. Here, these parameters were quantified using baseline intra-tumoral T cell concentration data stratified by cell surface markers. New methods supporting high throughput quantification of T cells result in an accumulation of data describing T cells within the tumor microenvironment (Gentles et al., 2015) or in the blood (Kagamu et al., 2020; Zhao et al., 2020; Lucca et al., 2021; Suh et al., 2021). The data can be used to predict the increase in CTLs after treatments either through machine learning methods or by incorporation into human QSP modeling of anti-PD-(L)1.

The identified parameters that contribute to inter-individual variability are for mice and it remains to be confirmed that the same parameters are relevant or most sensitive in humans. The local sensitivity analysis fell short of being able to investigate the combined impact of multiple parameters on treatment response. More sophisticated methods, such as virtual populations or machine learning methods, will be needed to investigate the predictive power of a subset of parameters. Fitting virtual populations to preclinical data may allow for incorporating more sources of variability into the parameter space while avoiding overfitting. Leveraging machine learning for parameter estimation or dimension reduction could help identify subsets of parameters or variables that determine treatment response (Zhang et al., 2022). Repeating parameter fitting for another cell line can support identified sources of variability or identify new inherent variability. The presented QSP framework can also be adjusted to describe other T cell populations of interest, such as regulatory or helper T cells or to model other drug treatments that target T cell activation, proliferation, and/or influx into the tumor microenvironment facilitating investigation of combination therapies.

In summary, the presented anti-PD-(L)1 QSP mouse model captures the variability inherent in the longitudinal tumor volume profiles through individual variability in the tumor cell proliferation rate, tumor carrying capacity, and CD8^+^ T cell proliferation half-life. We leveraged the model to investigate potential sources of variability in anti-PD-(L)1 mediated anti-tumor response and identified PD-(L)1 receptor expression, CD8^+^ T cell half-life, CTL-mediated tumor cell killing rate, and maximum rate of CD8^+^ T cell influx into the tumor microenvironment as having substantial impact on anti-tumor response. This model can be further translated to simulate clinical populations through reparameterization using published human PK and tumor properties. Inter-individual variability can be incorporated into first-in-human dose predictions through virtual population methods (Rieger et al., 2018; Rao et al., 2021).

## Data Availability

The raw data supporting the conclusion of this article will be made available by the authors, without undue reservation.

## Supplementary material

SUPPLEMENTARY FIGURE S1 PK fitting of literature data. Observed plasma concentrations (Zalba et al., 2020) after a single dose of 200 mg RMP1-14 and 100 mg 10F.9G2 (points) plotted with model simulations (lines).

SUPPLEMENTARY FIGURE S2 Tumor sizes at treatment start for each study.

SUPPLEMENTARY FIGURE S3 Longitudinal tumor volume profiles colored by observed rate of tumor volume change. The slow/responder category has an observed rate of tumor volume change between day 7 and day 13 after treatment start below 25 mm^3^ per day. All others are in the fast/non-responder category. The 25 mm^3^ per day threshold identifies individuals who experience complete tumor regression or a period of regression or stable growth.

SUPPLEMENTARY FIGURE S4 Longitudinal tumor volume profiles and model fittings for all studies. Observed tumor volumes for individual mice (colored dots) plotted with simulated individual profiles (colored lines) and the simulated population profile (black lines). Dosing times are shown by blue dashed lines. Details for the experimental setups are found in Table 1.

SUPPLEMENTARY FIGURE S5 Correlations between estimated individual parameters and observed rates of tumor volume change, stratified by study and treatment. Included is the Spearman rank correlation coefficient and *p*-value.**(A)** CD8+ T cell proliferation half-life, **(B)** tumor cell proliferation rate, and **(C)** tumor carrying capacity.

SUPPLEMENTARY FIGURE S6 Effect of PD-1 expression level on treatment response stratified by study. Percent change in tumor volume 12 days after treatment start for each fitted mouse with varying PD-1 or PD-L1 expression. Mice are arranged on the x-axis according to their estimated tumor cell proliferation rates. Treatment details are found in Table 1.

SUPPLEMENTARY FIGURE S7 Effect of PD-L1 expression level on treatment response stratified by study. Percent change in tumor volume 12 days after treatment start for each fitted mouse with varying PD-1 or PD-L1 expression. Mice are arranged on the x-axis according to their estimated tumor cell proliferation rates. Treatment details are found in Table 1.
