# Supplementary figures and images for: Sources of inter-individual variability leading to significant changes in anti-PD-1 and anti-PD-L1 efficacy identified in mouse tumor models using a QSP framework
Source: Front Pharmacol. 2022 Dec 5;13:1056365. doi: 10.3389/fphar.2022.1056365 (PMC9760747; doi:10.3389/fphar.2022.1056365)

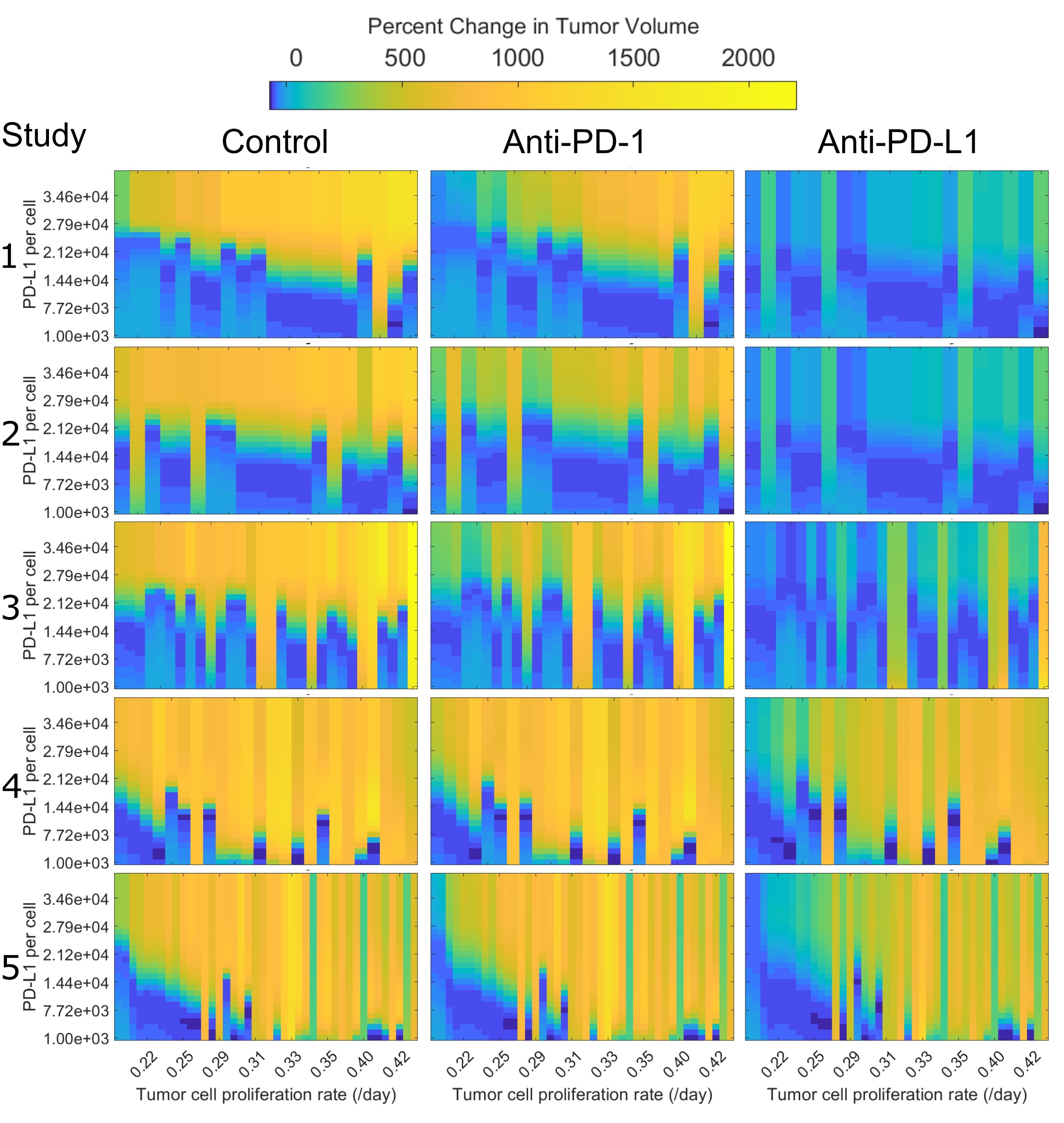

Supplement: Supplementary file 1 [file Image7.JPEG]

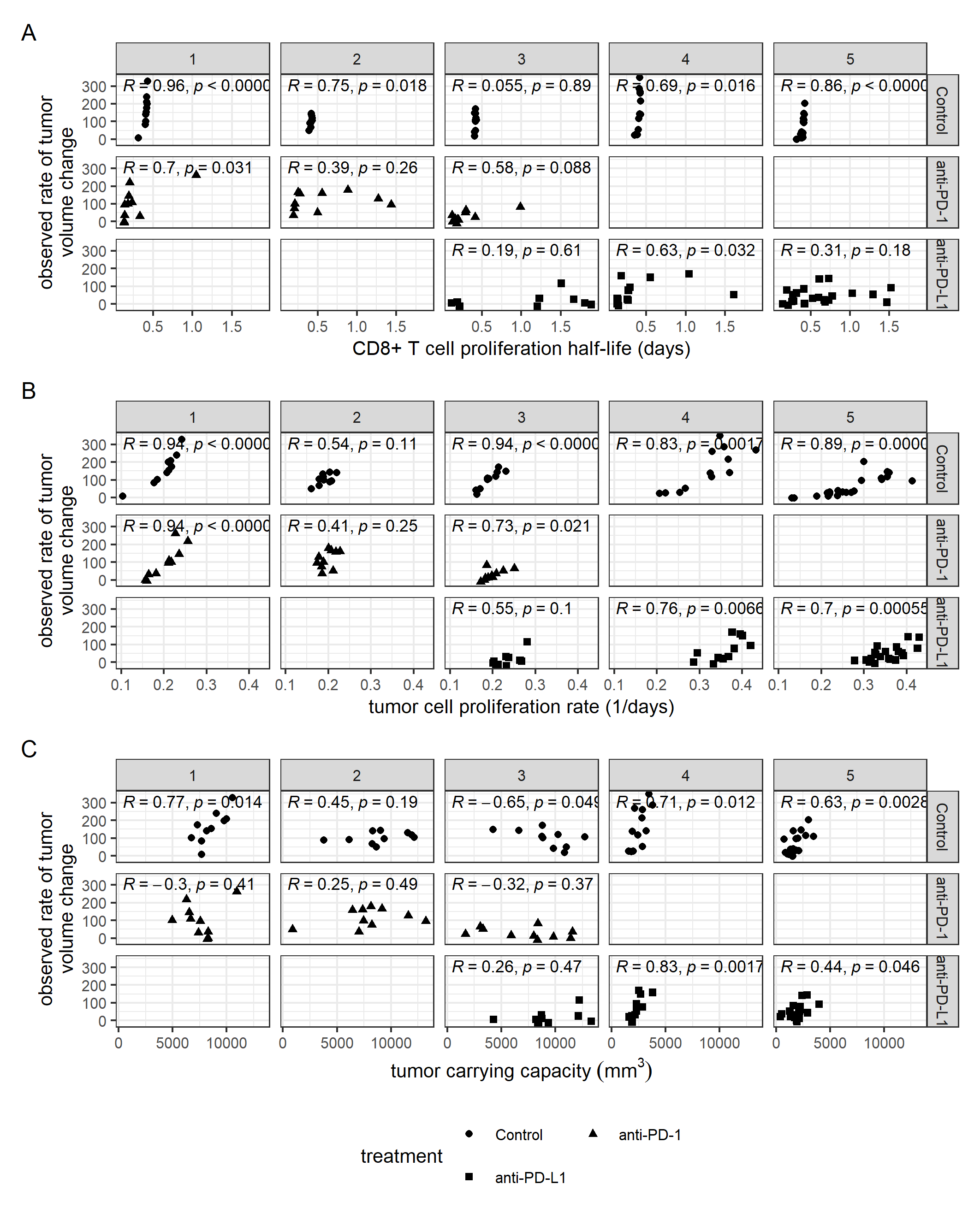

Supplement: Supplementary file 2 [file Image5.PNG]

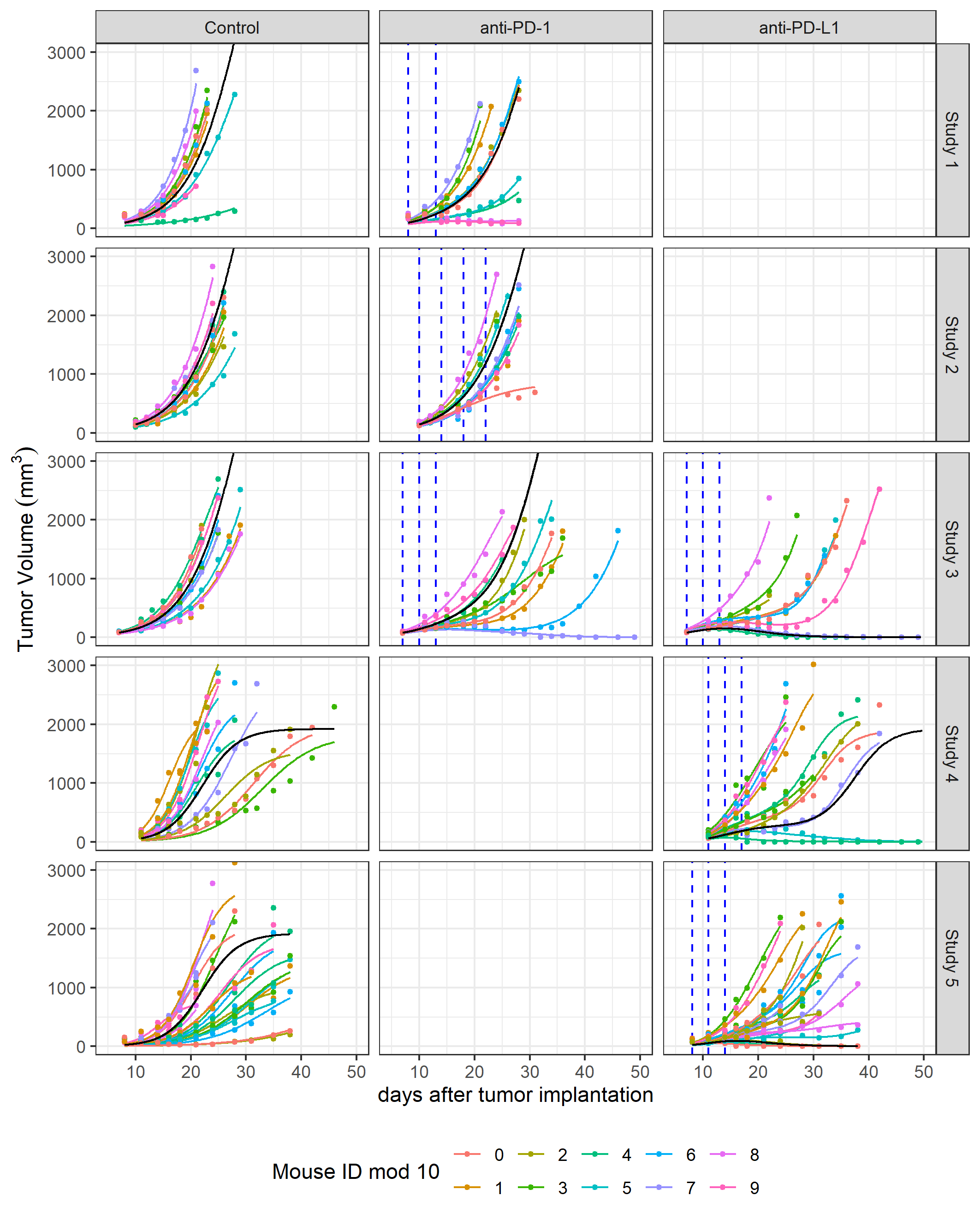

Supplement: Supplementary file 3 [file Image4.png]

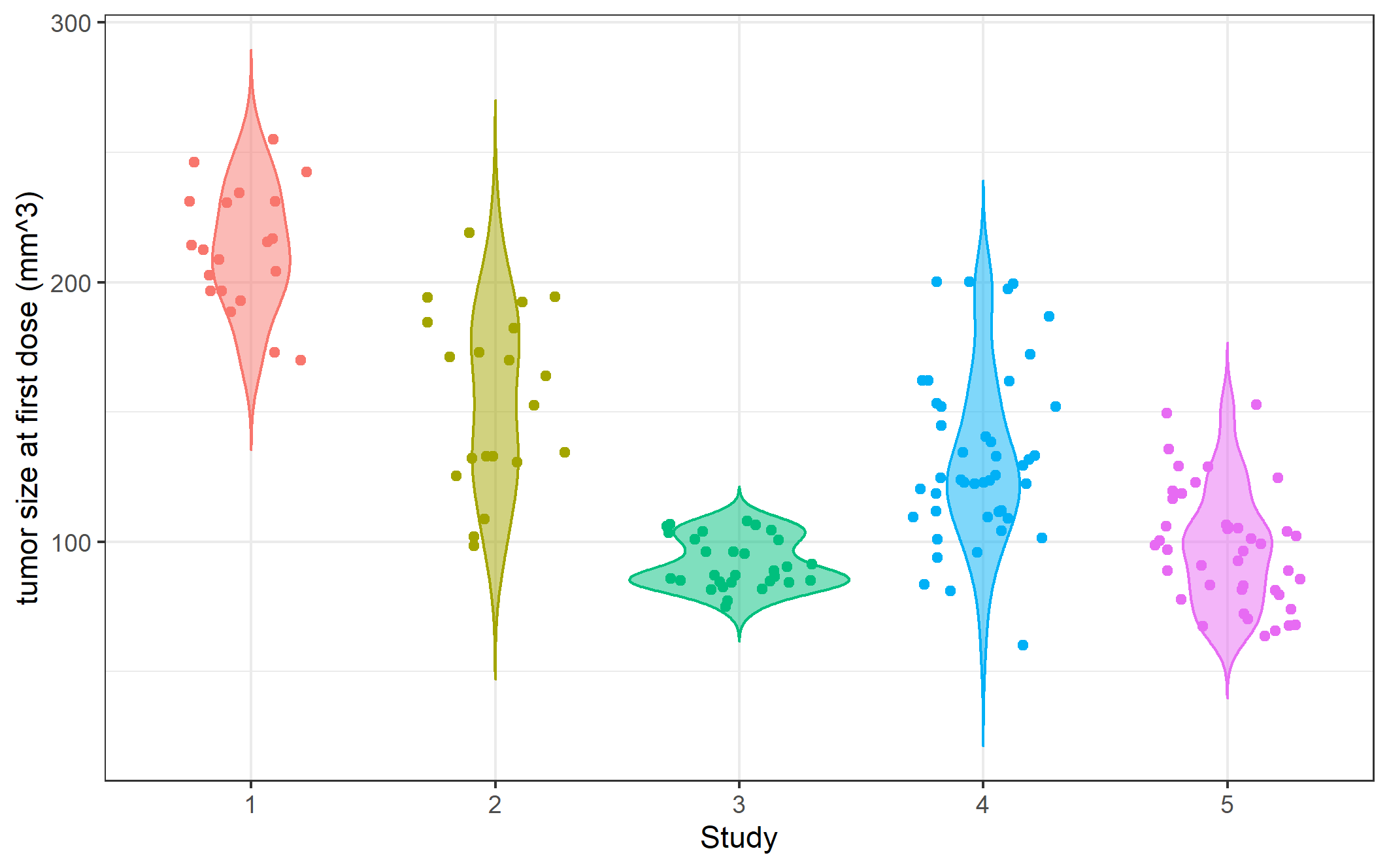

Supplement: Supplementary file 5 [file Image2.PNG]

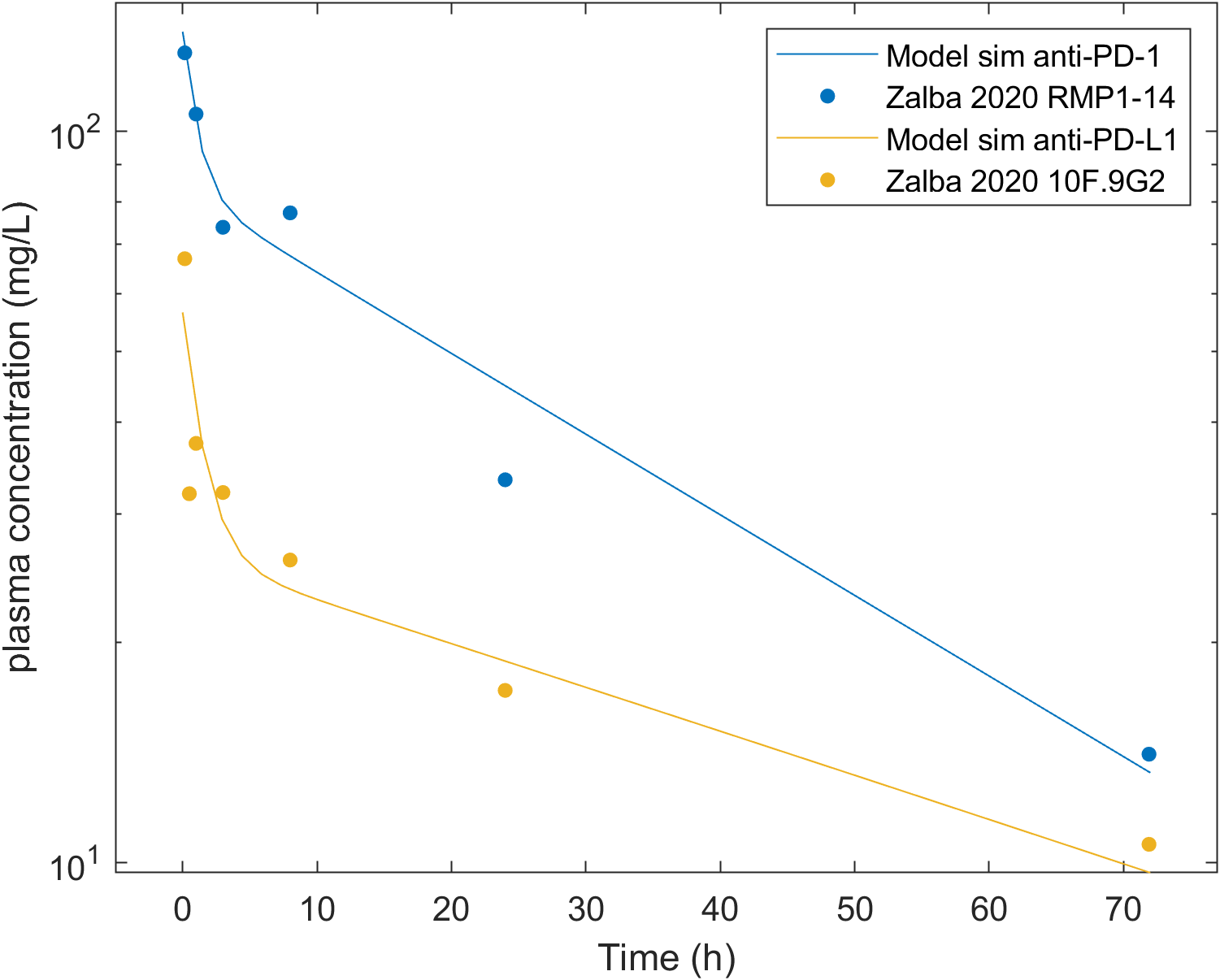

Supplement: Supplementary file 6 [file Image1.PNG]

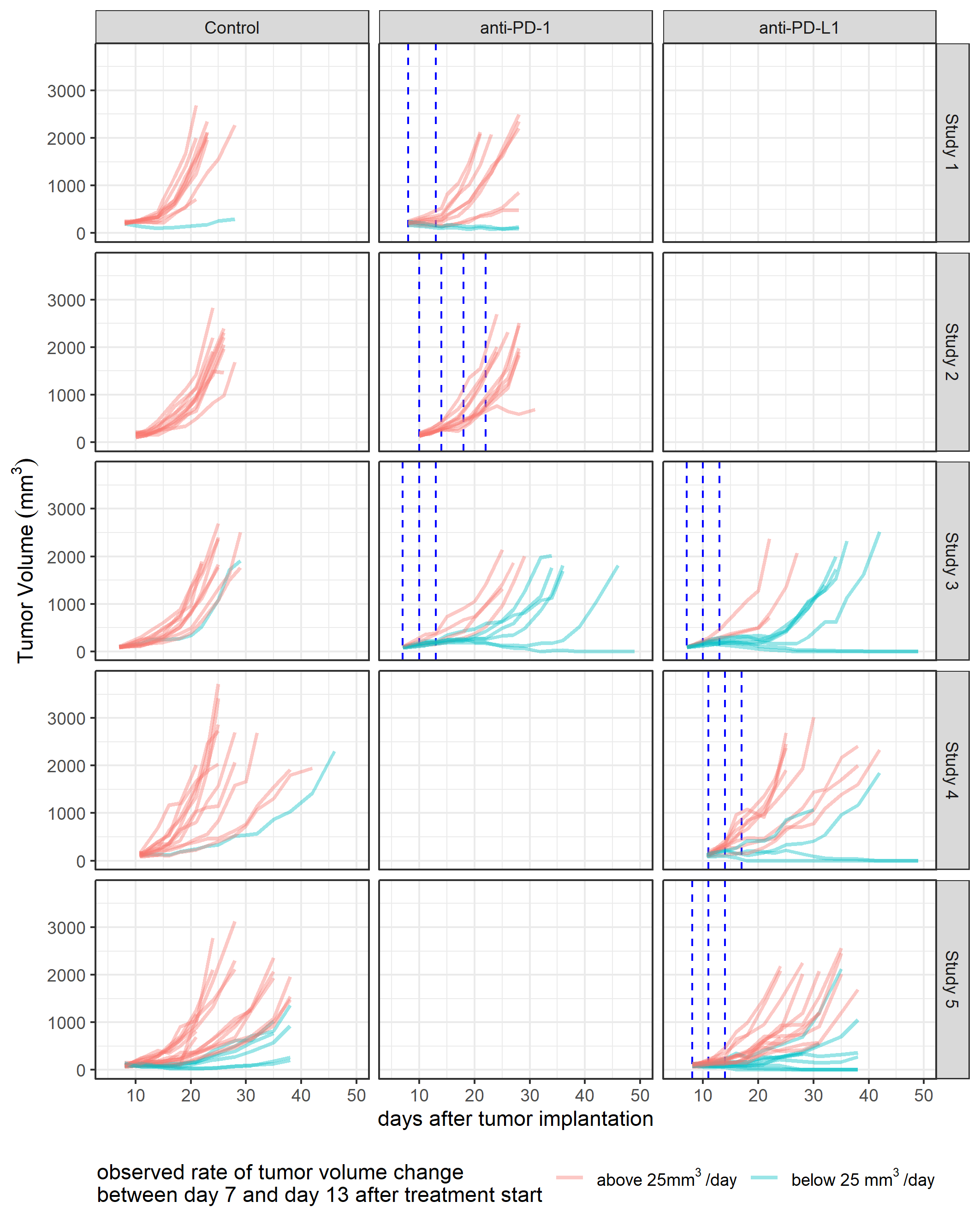

Supplement: Supplementary file 7 [file Image3.png]

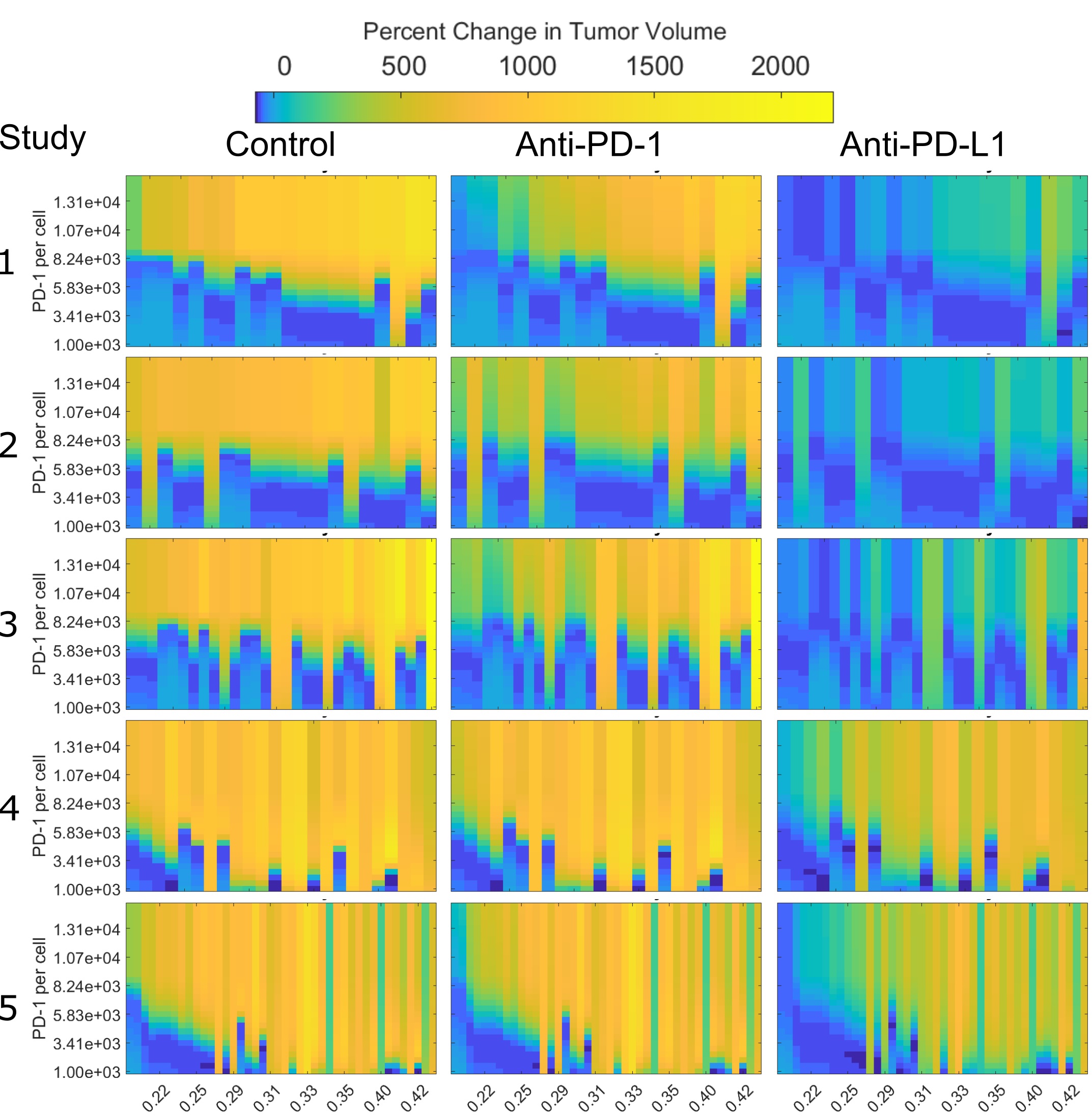

Supplement: Supplementary file 8 [file Image6.JPEG]
