# Supplementary material for: Sources of inter-individual variability leading to significant changes in anti-PD-1 and anti-PD-L1 efficacy identified in mouse tumor models using a QSP framework
Source: Front Pharmacol. 2022 Dec 5;13:1056365. doi: 10.3389/fphar.2022.1056365 (PMC9760747; doi:10.3389/fphar.2022.1056365)
Supplement: Supplementary file 4 [file DataSheet1.pdf]

Supplemental Information 1: Model Equations

QSP modeling was used to investigate sources of variability in anti-tumor response to anti-CTLA4 in CT26 syngeneic tumor bearing mice [7]. Herein, a QSP model of anti-PD-(L)1 is developed to understand the sources of variability in anti-tumor response in the same syngeneic mouse tumor model.

The QSP model consists of three compartments, including the plasma and peripheral compartments to capture the pharmacokinetics of anti-PD-(L)1 antibodies, and the tumor compartment to describe the pharmacodynamics within the tumor microenvironment.

See Table S1 for parameter and variable names, notations, and values.

1 Plasma (Central) Compartment

The plasma, or central, compartment is designated by the subscript c and describes the amount of anti-PD-1 (A_{c}^{P}), anti-PD-L1 (A_{c}^{L}), PD-1 (P_{c}), PD-L1 (L_{c}) and their bound complexes ($A{:}P_{\mathrm{c}}$, $A{:}L_{\mathrm{c}}$, $P{:}L_{\mathrm{c}}$) in the central compartment. PD-1 and PD-L1 are synthesized at the rates $k_{\mathrm{s,P,c}}$ and $k_{\mathrm{s,L,c}}$, respectively, and degrade at the rates $k_{\mathrm{d,P}}$ and $k_{\mathrm{d,L}}$. Membrane PD-1 and PD-L1 are present in the plasma compartment to capture the impact of potential target-mediated drug disposition on pharmacokinetics.

$$\begin{aligned}\frac{dP_{\mathrm{c}}}{dt} &= k_{\mathrm{s,P,c}} - k_{\mathrm{d,P}} P_{\mathrm{c}} \\ &\quad + k_{\mathrm{off,AP}} A{:}P_{\mathrm{c}} - k_{\mathrm{onAP}} A_{\mathrm{c}}^{P} P_{\mathrm{c}}/V_{\mathrm{c}} \\ &\quad + k_{\mathrm{off,PL}} P{:}L_{\mathrm{c}} - k_{\mathrm{on,PL}} P_{\mathrm{c}} L_{\mathrm{c}}/V_{\mathrm{c}}\end{aligned} \tag{1}$$

$$\begin{aligned}\frac{dL_{\mathrm{c}}}{dt} &= k_{\mathrm{s,L,c}} - k_{\mathrm{d,L}} L_{\mathrm{c}} \\ &\quad + k_{\mathrm{off,AL}} A{:}L_{\mathrm{c}} - k_{\mathrm{on,AL}} A_{\mathrm{c}}^{L} L_{\mathrm{c}}/V_{\mathrm{c}} \\ &\quad + k_{\mathrm{off,PL}} P{:}L_{\mathrm{c}} - k_{\mathrm{on,PL}} P_{\mathrm{c}} L_{\mathrm{c}}/V_{\mathrm{c}}\end{aligned} \tag{2}$$

$$\frac{dP{:}L_{\mathrm{c}}}{dt} = -\,k_{\mathrm{off,PL}} P{:}L_{\mathrm{c}} + k_{\mathrm{on,PL}} P_{\mathrm{c}} L_{\mathrm{c}}/V_{\mathrm{c}} - k_{\mathrm{d,PL}} P{:}L_{\mathrm{c}} \tag{3}$$

The antibodies are cleared from the bloodstream at the rates $k_{\mathrm{el,A^P}}$ and $k_{\mathrm{el,A^L}}$ for anti-PD-1 and anti-PD-L1 respectively. Distribution to the peripheral compartment occurs at rates $k_{\mathrm{cp,A^P}}$ and $k_{\mathrm{cp,A^L}}$ while flow back from the peripheral into the central compartment occurs at rates $k_{\mathrm{pc,A^P}}$ and $k_{\mathrm{pc,A^L}}$. Flux between the central and tumor compartments

($k_{\mathrm{ct,A^P}}$ and $k_{\mathrm{ct,A^L}}$) are described in Section 1.1 and is based on the interstitial tumor volume ($V_{\mathrm{t}}\epsilon$).

$$\begin{aligned}
\frac{dA_{\mathrm{c}}^{P}}{dt} =& -k_{\mathrm{el,A^P}} A_{\mathrm{c}}^{P} \\
&+ k_{\mathrm{off,AP}} A{:}P_{\mathrm{c}} - k_{\mathrm{on,AP}} A_{\mathrm{c}}^{P} P_{\mathrm{c}}/V_{\mathrm{c}} \\
&+ k_{\mathrm{pc,A^P}} A_{\mathrm{p}}^{P} - k_{\mathrm{cp,A^P}} A_{\mathrm{c}}^{P} \\
&- k_{\mathrm{ct,A^P}} V_{\mathrm{t}}\epsilon
\end{aligned} \tag{4}$$

$$\frac{dA{:}P_{\mathrm{c}}}{dt} = k_{\mathrm{on,AP}} A_{\mathrm{c}}^{P} P_{\mathrm{c}}/V_{\mathrm{c}} - k_{\mathrm{off,AP}} A{:}P_{\mathrm{c}} - k_{\mathrm{d,AP}} A{:}P_{\mathrm{c}} \tag{5}$$

$$\begin{aligned}
\frac{dA_{\mathrm{c}}^{L}}{dt} =& -k_{\mathrm{el,A^L}} A_{\mathrm{c}}^{L} \\
&+ k_{\mathrm{off,AL}} A{:}L_{\mathrm{c}} - k_{\mathrm{on,AL}} A_{\mathrm{c}}^{L} L_{\mathrm{c}}/V_{\mathrm{c}} \\
&+ k_{\mathrm{pc,A^L}} A_{\mathrm{p}}^{L} - k_{\mathrm{cp,A^L}} A_{\mathrm{c}}^{L} \\
&- k_{\mathrm{ct,A^L}} V_{\mathrm{t}}\epsilon
\end{aligned} \tag{6}$$

$$\frac{dA{:}L_{\mathrm{c}}}{dt} = k_{\mathrm{on,AL}} A_{\mathrm{c}}^{L} L_{\mathrm{c}}/V_{\mathrm{c}} - k_{\mathrm{off,AL}} A{:}L_{\mathrm{c}} - k_{\mathrm{d,AL}} A{:}L_{\mathrm{c}} \tag{7}$$

1.1 Transfer to tumor compartment

Antibody distribution to the tumor is based on the work of Li et al. [6] which models movement of the antibodies as a function of the permeability of the capillary and diffusion into the tumor tissue. The capillary radius (R_{cap}), the average radius of tissue surrounding each blood vessel (R_{Krogh}), and the permeability constant ($P_{\mathrm{A^P}}$) determine movement through the capillary membrane while the diffusion constant ($D_{\mathrm{A^P}}$) and the tumor radius (R_{tumor}) determine the rate of diffusion. Both permeability and diffusion constants are estimated based on the molecular weights of a bivalent antibody of 150 kDa [6]. The fraction of tumor volume that is outside of cells (the intersitial volume) is denoted by ϵ.

$$k_{\mathrm{ct,A^P}} = \frac{2P_{\mathrm{A^P}} R_{\mathrm{cap}}}{R_{\mathrm{Krogh}}^2} \left(\frac{A_{\mathrm{c}}^{P}}{V_{\mathrm{c}}} - \frac{A_{\mathrm{t}}^{P}}{V_{\mathrm{t}}\epsilon} \right) + \frac{6D_{\mathrm{A^P}}}{R_{\mathrm{tumor}}^2} \left(\frac{A_{\mathrm{c}}^{P}}{V_{\mathrm{c}}} - \frac{A_{\mathrm{t}}^{P}}{V_{\mathrm{t}}\epsilon} \right) \tag{8}$$

$$k_{\mathrm{ct,A^L}} = \frac{2P_{\mathrm{A^L}} R_{\mathrm{cap}}}{R_{\mathrm{Krogh}}^2} \left(\frac{A_{\mathrm{c}}^{L}}{V_{\mathrm{c}}} - \frac{A_{\mathrm{t}}^{L}}{V_{\mathrm{t}}\epsilon} \right) + \frac{6D_{\mathrm{A^L}}}{R_{\mathrm{tumor}}^2} \left(\frac{A_{\mathrm{c}}^{L}}{V_{\mathrm{c}}} - \frac{A_{\mathrm{t}}^{L}}{V_{\mathrm{t}}\epsilon} \right) \tag{9}$$

$$P_{\mathrm{A^P}} = P_{\mathrm{A^L}} = 8 \times 10^{-6} \times 150^{-0.553} \qquad [6] \tag{10}$$

$$D_{\mathrm{A^P}} = D_{\mathrm{A^L}} = 1 \times 10^{-5} \times 150^{-0.925} \qquad [6] \tag{11}$$

2 Peripheral Compartment

Antibody amounts in the peripheral compartment (denoted with a p subscript) are purely functions of influx from and outflow to the central compartment. No synthesis

or degradation is modeled.

$$\frac{dA_{\mathrm{p}}^{P}}{dt} = -k_{\mathrm{pc,A^P}} A_{\mathrm{p}}^{P} + k_{\mathrm{cp,A^P}} A_{\mathrm{c}}^{P} \tag{12}$$

$$\frac{dA_{\mathrm{p}}^{L}}{dt} = -k_{\mathrm{pc,A^L}} A_{\mathrm{p}}^{L} + k_{\mathrm{cp,A^L}} A_{\mathrm{c}}^{L} \tag{13}$$

3 Tumor Compartment

We assume the tumor is well mixed and that T cell concentrations are constant as tumor volume changes. The fraction of tumor volume not comprised of cells (the interstitial volume) is ϵ.

3.1 Cells

In the tumor compartment the concentrations of tumor cells (T^i), PD-1+CD8+ T cells (E^i), and other PD-1+ T cells (H) were modeled.

Tumor growth is modeled using a logistic growth curve due to its ability to describe tumor growth past the initial stage of exponential growth [5] and for consistency with previous mouse tumor modeling publications [2, 7] with growth rate $k_{\mathrm{pfr,T}}$ and carrying capacity $k_{\mathrm{limit,T}}$. Tumor cells (T^V) grow according to this curve until they are damaged either through ADCC ($f_{\mathrm{ADCC}}()$) due to binding with anti-PD-L1 or through interaction with a CTL with rate k_{kill}. Damaged tumor cells (T^M) undergo apoptosis with rate k_{apop}. The total tumor volume (V_{t}) is the sum of the undamaged and damaged tumor cells divided by the constant tumor cell concentration (Eq. 16). Additional volume from immune cells is assumed negligible due to their smaller size and lower concentration (5-7 um, 2.15×10^2 cells per mm^3, vs. 12-25 um [4], 10^8 cells per mm^3 [1] for tumor cells). For numerical stability, a minimum tumor size of 1 mm^3 was implemented.

$$\frac{dT^V}{dt} = T^V k_{\mathrm{pfr,T}}(1 - \frac{T^V}{k_{\mathrm{limit,T}}}) - f_{\mathrm{ADCC}}\left(\frac{A{:}L^V}{T^V}\right) T^V - k_{\mathrm{kill}}(\sum_{i=1}^{10} E^{D_i}) T^V / V_{\mathrm{t}} \tag{14}$$

$$\frac{dT^M}{dt} = k_{\mathrm{kill}}(\sum_{i=1}^{10} E^{D_i}) T^V / V_{\mathrm{t}} + f_{\mathrm{ADCC}}\left(\frac{A{:}L^V}{T^V}\right) T^V - k_{\mathrm{apop}} T^M \tag{15}$$

$$V_t = (T^V + T^M)/C_{TC} \tag{16}$$

Anti-PD-L1 molecules capable of ADCC damage tumor cells according to the number bound per tumor cell ($\frac{A:L^V}{T^V}$) with a maximum rate of $E_{\mathrm{max}}^{\mathrm{ADCC}}$ and achieving half that rate at $EC50_{\mathrm{ADCC}}$.

$$f_{\mathrm{ADCC}}\left(\frac{A{:}L^V}{T^V}\right) = \frac{E_{\mathrm{max}}^{\mathrm{ADCC}} \frac{A:L^V}{T^V}}{EC50_{\mathrm{ADCC}} + \frac{A:L^V}{T^V}} \tag{17}$$

Other PD-1 positive CD8+ T cells (H) cells serve only as a source of PD-1 to competitively bind with PD-L1 on tumor cells, and are synthesized at the rate $k_{\mathrm{s,H}}$ and degrade at the rate $k_{d,H}$. We assume that the immune cell concentration is constant as the tumor volume changes, as such the synthesis rate is multiplied by the tumor volume (V_{t}).

$$\frac{dH}{dt} = k_{\mathrm{s,H}} V_t - k_{d,H} H \tag{18}$$

Inside the tumor compartment, PD-1+CD8+ T cells are synthesized as inactive cells (E^I). The cells go through eight proliferation stages (E^A, E^{P_i} for $i = 1, ..., 7$) before becoming CTLs (E^{D_i} for $i = 1, ..., 10$) [7]. Eight proliferation stages was chosen to match the magnitude of T cell expansion seen in [8]. Each CTL can damage ten tumor cells before becoming exhausted (E^{D_0}) [3]. Inactive, active, and proliferating CD8+ T cells degrade at a rate of $k_{\mathrm{d,CD8}}$ while CTLs degrade at a rate of $k_{d,CTL}$.

The binding of PD-L1 expressed on tumor cells to PD-1 expressed on inactive PD-1+CD8+ T cells ($P^I : L^V$) controls T cell behavior. This binding inhibits the influx (f_{influx}) of inactive PD-1+CD8+ T cells into the tumor microenvironment, and the activation (f_{activate}) of PD-1+CD8+ T cells in the tumor compartment. In the absence of antibody treatments PD-1:PD-L1 per inactive CD8+ T cell is at its steady state concentrations (approximately 7.1) causing inactive PD-1+CD8+ T cells to enter the tumor compartment at the rate of $k_{s,I} V_t$ (Eq. 56). This influx rate ensures that the CD8+T cells reach a steady state concentration during control simulations.

Both the activation and influx functions reach their maximum (E^i_{max}) when there is no bound PD-1:PD-L1. As the bound per inactive T cell increases, the activation and influx decreases. Since both the activation and influx functions are dependent on PD-1:PD-L1 binding on inactive CD8 cells, we use the same EC50 and Hill coefficient (c=1) for both.

$$\begin{aligned} \frac{dE^I}{dt} = & f_{\mathrm{influx}}\left(\frac{P^I{:}L^V}{E^I}\right) - k_{\mathrm{d,CD8}} E^I \\ & - f_{\mathrm{activate}}\left(\frac{P^I{:}L^V}{E^I}\right) E^I + k_{\mathrm{A2Ibasal}} E^A \end{aligned} \tag{19}$$

$$f_{\mathrm{influx}}\left(\frac{P^I{:}L^V}{E^I}\right) = \max\left[0, \frac{E^{\mathrm{influx}}_{\mathrm{max}}}{1+\left(\frac{EC50}{6.6}\right)^c} - \frac{E^{\mathrm{influx}}_{\mathrm{max}}}{1+\left(\frac{EC50}{\frac{P^I:L^V}{E^I}}\right)^c}\right] + k_{\mathrm{s,I}} V_{\mathrm{t}} \tag{20}$$

$$f_{\mathrm{activate}}\left(\frac{P^I{:}L^V}{E^I}\right) = E^{\mathrm{activate}}_{\mathrm{max}} - \frac{E^{\mathrm{activate}}_{\mathrm{max}}}{1+\left(\frac{EC50}{\frac{P^I:L^V}{E^I}}\right)^c} \tag{21}$$

In the presented model set up, during control simulations, nearly all tumor cells are undamaged and the inactive CD8+ T cell concentration converges to steady state value from data (section 4) leading to a consistent value for $\frac{P^I:L^V}{E^I}$ while fitting the remaining parameters. While for each individual the exact receptor occupancy per inactive CD8+ T cell may not be 6.6, the value works well for maintaining influx equal to $k_{\mathrm{s,I}}V_{\mathrm{t}}$ at baseline and higher when receptor occupancy drops with treatment.

Activated CD8+PD-1+ T cells (E^A) follow the same activation rates as described above. Cells deactivate at the rate k_{A2Ibasal} and double with rate k_{pfr}; for each cell leaving E^A, two cells enter E^{P_1}.

$$\begin{aligned}\frac{dE^A}{dt} = f_{\mathrm{activate}}\left(\frac{P^I:L^V}{E^I}\right)E^I - k_{\mathrm{d,CD8}}E^A \\ - k_{\mathrm{A2Ibasal}}E^A - k_{\mathrm{pfr}}E^A\end{aligned} \tag{22}$$

The cells continue proliferating, passing through seven stages (E_{P_i} for $i = 1, ..., 7$).

$$\frac{dE^{P_1}}{dt} = 2k_{\mathrm{pfr}}E^A - k_{\mathrm{pfr}}E^{P_1} - k_{\mathrm{d,CD8}}E^{P_1} \tag{23}$$

$$\frac{dE^{P_i}}{dt} = 2k_{\mathrm{pfr}}E^{P_{i-1}} - k_{\mathrm{pfr}}E^{P_i} - k_{\mathrm{d,CD8}}E^{P_i}, \qquad \text{for } i = 2, ..., 7 \tag{24}$$

After proliferating, the cells are now cytotoxic lymphocytes and have the ability to damage tumor cells. Every time a cell damages a tumor cell it moves from stage E^{D_i} to $E^{D_{i-1}}$. In stage E^{D_0}, the cell is considered exhausted, cannot damage any tumor cells, and is left to degrade. CTLs damage tumor cells at the rate k_{kill}.

$$\frac{dE^{D_{10}}}{dt} = 2k_{\mathrm{pfr}}E^{P_7} - k_{\mathrm{d,CTL}}E^{D_{10}} - k_{\mathrm{kill}}E^{D_{10}}T^V/V_{\mathrm{t}} \tag{25}$$

$$\frac{dE^{D_i}}{dt} = k_{\mathrm{kill}}E^{D_{i+1}}T^V/V_{\mathrm{t}} - k_{\mathrm{d,CTL}}E^{D_i} - k_{\mathrm{kill}}E^{D_i}T^V/V_{\mathrm{t}}, \qquad \text{for } i = 9, ..., 1 \tag{26}$$

$$\frac{dE^{D_0}}{dt} = k_{\mathrm{kill}}E^{D_1}T^V/V_{\mathrm{t}} - k_{\mathrm{d,CTL}}E^{D_0} \tag{27}$$

3.2 Free Antibodies

Antibody amounts in the tumor (denoted with a t subscript) are dependent on flux from the central compartment (see Section 1.1) and binding to and unbinding from receptors on the various cells within the tumor compartment at rates $k_{\mathrm{on,AP}}$, and $k_{\mathrm{off,AP}}$ for anti-PD-1 and $k_{\mathrm{on,AL}}$, and $k_{\mathrm{off,AL}}$ for anti-PD-L1. PD-1 on H or E cells are denoted P^i for $i \in \{H, I, A, P, D\}$ while PD-L1 on tumor cells are denoted L^V and L^M for tumor cells and damaged tumor cells respectively.

No degradation of free antibodies happens within the tumor compartment, but antibodies bound to receptors on cells can be internalized (described in Section 3.5). Antibodies bound to cells are denoted as $A{:}P^i$ for $i \in \{H, I, A, P, D\}$ for anti-PD-1 bound

to H or E cells while $A{:}L^V$ and $A{:}L^M$ denote anti-PD-L1 to PD-L1 complexes on tumor cells and damaged tumor cells, respectively.

$$\begin{aligned}\frac{dA_{\mathrm{t}}^P}{dt} &= k_{\mathrm{off,AP}}(A{:}P^H + A{:}P^I + A{:}P^A + A{:}P^P + A{:}P^D) \\ &\quad - k_{\mathrm{on,AP}}A_{\mathrm{t}}^P(P^H + P^I + P^A + P^P + P^D)/(V_{\mathrm{t}}\epsilon) \\ &\quad + k_{\mathrm{ct,A^P}}V_{\mathrm{t}}\epsilon\end{aligned} \tag{28}$$

$$\begin{aligned}\frac{dA_{\mathrm{t}}^L}{dt} &= k_{\mathrm{off,AL}}(A{:}L^V + A{:}L^M) \\ &\quad - k_{\mathrm{on,AL}}A_{\mathrm{t}}^L(L^V + L^M)/(V_{\mathrm{t}}\epsilon) \\ &\quad + k_{\mathrm{ct,A^L}}V_{\mathrm{t}}\epsilon\end{aligned} \tag{29}$$

3.3 PD-1 Receptors

PD-1 receptors on cells are tracked according to the following assumptions:

- New cells are created with the average number of surface receptors per cell (S_{H}, S_{E} for H and E cells respectively).
- PD-1 receptors degrade at the rate $k_{\mathrm{d,P}}$.
- Cells synthesize PD-1 at a rate $k_{\mathrm{s,P,H}} = S_{\mathrm{H}} \times k_{\mathrm{d,P}}$ and $k_{\mathrm{s,P,CD8}} = S_{\mathrm{E}} \times k_{\mathrm{d,P}}$ for H and E cells, respectively.
- When a cell degrades, all receptors on the cell are eliminated.
- When a tumor cell that is bound to an PD-1+ cells dies (through apoptosis or the death term from the logistic growth equation), the PD-1 receptors return to the unbound pool.
- To minimize the number of equations, PD-1 receptors on proliferating (E^{P_i}) and CTL (E^{D_i}) cells are gathered into their respective pools (P^P and P^D). The PD-1 receptors are assumed to be evenly distributed between the cells in the group.
- When a cell moves one stage downstream (for example, E^{P_7} to $E^{D_{10}}$), the receptors on the cell also move one stage downstream.

Comments are provided for the first two equations to explain each term.

$$\frac{dP^H}{dt} = k_{\mathrm{s,P,H}} \times H \quad \text{Synthesis of new receptors on } H \text{ cells} \tag{30}$$

$$+ k_{\mathrm{s,H}} \times S_{\mathrm{H}} \times V_{\mathrm{t}} \quad \text{Synthesis of new } H \text{ cells with average number of PD-1}$$

$$- k_{\mathrm{d,H}} \times S_{\mathrm{H}} P^H \quad \text{degradation of } H \text{ cells}$$

$$- k_{\mathrm{d,P}} P^H \quad \text{degradation of PD-1 on } H \text{ cells}$$

$$+ k_{\mathrm{off,AP}} A{:}P^H \quad \text{degradation of PD-1 on } H \text{ cells}$$

$$- k_{\mathrm{on,AP}} A^P P^H/(V_{\mathrm{t}}\epsilon) \quad \text{binding to anti-PD-1}$$

$$+ k_{\mathrm{off,PL}}(P^H{:}L^V + P^H{:}L^M) \quad \text{unbinding from PD-L1 on tumor cells}$$

$$- k_{\mathrm{on,PL}}(P^H L^V + P^H L^M)/(V_{\mathrm{t}}\epsilon) \quad \text{Binding to PD-L1 on tumor cells}$$

$$+ k_{\mathrm{apop}} P^H{:}L^M \quad \text{PD-1 that was bound to PD-L1 on damaged tumor cells that died from apoptosis}$$

$$+ k_{\mathrm{pfr,T}}/k_{\mathrm{limit,T}} P^H{:}L^V \times T^V \quad \text{PD-1 that was bound to PD-L1 on tumor cells that died from logistic eq}$$

$$\frac{dP^I}{dt} = k_{\mathrm{s,P,CD8}} E^I \quad \text{Synthesis PD-1 on } E^I \tag{31}$$

$$+ f_{\mathrm{influx}}\left(\frac{P^I{:}L^V}{T^V}\right) \quad \text{Influx of } E^I \text{ with average PD-1 per cell}$$

$$- k_{\mathrm{d,CD8}} P^I \quad \text{degradation of } E^I$$

$$- k_{\mathrm{d,P}} P^I \quad \text{degradation of PD-1 on } E^I$$

$$+ k_{\mathrm{off,PL}}(P^I{:}L^V + P^I{:}L^M) \quad \text{unbinding from PD-L1 on tumor cells}$$

$$- k_{\mathrm{on,PL}}(P^I \times L^V + P^I \times L^M)/(V_{\mathrm{t}}\epsilon) \quad \text{binding to PD-L1 on tumor cells}$$

$$+ k_{\mathrm{off,AP}} A{:}P^I - k_{\mathrm{on,AP}} A^P \times P^I/(V_{\mathrm{t}}\epsilon) \quad \text{binding to anti-PD-1}$$

$$+ k_{\mathrm{A2Ibasal}} P^A - \quad \text{basal deactivation of } E^I$$

$$f_{\mathrm{activate}}\left(\frac{P^I{:}L^V}{E^I}\right) P^I \quad \text{activation of } E^I$$

$$+ k_{\mathrm{apop}} P^I{:}L^M \quad \text{PD-1 that was bound to damaged tumor cells tumor cells that died from apoptosis}$$

$$+ k_{\mathrm{pfr,T}}/k_{\mathrm{limit,T}} P^I{:}L^V \times T^V \quad \text{PD-1 that was bound to PD-L1 on tumor cells that died from logistic eq}$$

$$\begin{aligned}
\frac{dP^A}{dt} &= k_{\text{s,P,CD8}}E^A - k_{\text{d,CD8}}P^A - k_{\text{d,P}}P^A \\
&- k_{\text{A2Ibasal}}P^A + f_{\text{activate}}\left(\frac{P^I{:}L^V}{E^I}\right)P^I \\
&+ k_{\text{off,PL}}(P^A{:}L^V + P^A{:}L^M) - k_{\text{on,PL}}(P^AL^V + P^AL^M)/(V_{\text{t}}\epsilon) \\
&+ k_{\text{off,AP}}A{:}P^A - k_{\text{on,AP}}A^PP^A/(V_{\text{t}}\epsilon) \\
&- k_{\text{pfrCD8}}P^A \\
&+ k_{\text{apop}}P^A{:}L^M + k_{\text{pfr,T}}/k_{\text{limit,T}}P^A{:}L^V \times T^V
\end{aligned} \tag{32}$$

$$\begin{aligned}
\frac{dP^P}{dt} &= k_{\text{s,P,CD8}}\sum_{i=1}^{7}E^{P_i} - k_{\text{d,CD8}}P^P - k_{\text{d,P}}P^P \\
&+ k_{\text{off,PL}}(P^P{:}L^V + P^P{:}L^M) - k_{\text{on,PL}}(P^PL^V + P^PL^M)/(V_{\text{t}}\epsilon) \\
&+ k_{\text{off,AP}}A{:}P^P - k_{\text{on,AP}}A^PP^P/(V_{\text{t}}\epsilon) \\
&+ k_{\text{pfrCD8}}(2P^A + A{:}P^A + P^A{:}L^V + P^A{:}L^M) \\
&+ k_{\text{pfrCD8}}(P^P + A{:}P^P + P^P{:}L^V + P^P{:}L^M)(1 - \frac{E^{P_7}}{\sum_{i=1}^{7}E^{P_i}}) \\
&- k_{\text{pfrCD8}}(P^P + A{:}P^P + P^P{:}L^V + P^P{:}L^M)\frac{E^{P_7}}{\sum_{i=1}^{7}E^{P_i}} \\
&+ k_{\text{apop}}P^P{:}L^M + k_{\text{pfr,T}}/k_{\text{limit,T}}P^P{:}L^V \times T^V
\end{aligned} \tag{33}$$

$$\begin{aligned}
\frac{dP^D}{dt} &= k_{\text{s,P,CD8}}\sum_{i=0}^{10}E^{D_i} - k_{\text{d,CTL}}P^D - k_{\text{d,P}}P^D \\
&+ k_{\text{pfrCD8}}(2P^P + A{:}P^P + P^P{:}L^V + P^P{:}L^M)\frac{E^{P_7}}{\sum_{i=1}^{7}E^{P_i}} \\
&+ k_{\text{off,PL}}(P^D{:}L^V + P^D{:}L^M) - k_{\text{on,PL}}(P^DL^V + P^DL^M)/(V_{\text{t}}\epsilon) \\
&+ k_{\text{off,AP}}A{:}P^D - k_{\text{on,AP}}A^PP^D/(V_{\text{t}}\epsilon) \\
&+ k_{\text{apop}}P^D{:}L^M + k_{\text{pfr,T}}/k_{\text{limit,T}}P^D{:}L^V \times T^V
\end{aligned} \tag{34}$$

3.4 PD-L1 Receptors

PD-L1 receptors on tumor cells are tracked according to the following assumptions:

- New cells are created with the average number of receptors per cell (S_{TC})
- PD-L1 receptors degrade at the rate $k_{\text{d,L}}$
- Tumor cells synthesize PD-L1 at a rate $k_{\text{s,L}} = S_{TC} \times k_{\text{d,L}}$
- When a cell degrades, all the receptors on the cell are eliminated

- When a PD-1+ cell that is bound to a tumor cell dies, the PD-L1 receptors return to the unbound pool

$$
\begin{aligned}
\frac{dL^V}{dt} &= k_{\mathrm{pfr,T}}(L^V + A{:}L^V) - \frac{k_{\mathrm{pfr,T}}}{k_{\mathrm{limit,T}}} T^V L^V + k_{\mathrm{s,L}} T^V - k_{\mathrm{d,L}} L^V \\
&+ k_{\mathrm{off,PL}}(P^H{:}L^V + P^I{:}L^V + P^A{:}L^V + P^P{:}L^V + P^D{:}L^V) \\
&- k_{\mathrm{on,PL}} L^V (P^H + P^I + P^A + P^P + P^D)/(V_{\mathrm{t}}\epsilon) \\
&+ k_{\mathrm{off,AL}} A{:}L^V - k_{\mathrm{on,AL}} A^L L^V/(V_{\mathrm{t}}\epsilon) \\
&- k_{\mathrm{kill}} L^V (\sum_{i=1}^{10} E^{D_i})/V_{\mathrm{t}} \\
&- f_{\mathrm{ADCC}} \left(\frac{A{:}L^V}{T^V}\right) L^V \\
&+ k_{\mathrm{d,H}} P^H{:}L^V \\
&+ k_{\mathrm{d,CD8}}(P^I{:}L^V + P^A{:}L^V + P^P{:}L^V) + +k_{\mathrm{d,CD8}} P^D{:}L^V
\end{aligned} \tag{35}
$$

$$
\begin{aligned}
\frac{dL^M}{dt} &= k_{\mathrm{s,L}} T^M - k_{\mathrm{d,L}} L^M - k_{\mathrm{apop}} L^M \\
&+ k_{\mathrm{off,PL}}(P^H{:}L^M + P^I{:}L^M + P^A{:}L^M + P^P{:}L^M + P^D{:}L^M) \\
&- k_{\mathrm{on,PL}} L^M (P^H + P^I + P^A + P^P + P^D)/(V_{\mathrm{t}}\epsilon) \\
&+ k_{\mathrm{off,AL}} A{:}L^M - k_{\mathrm{on,AL}} A^L L^M/(V_{\mathrm{t}}\epsilon) \\
&+ k_{\mathrm{kill}} L^V (\sum_{i=1}^{10} E^{D_i})/V_{\mathrm{t}} \\
&+ f_{\mathrm{ADCC}} \left(\frac{A{:}L^V}{T^V}\right) L^V \\
&+ k_{\mathrm{d,H}} P^H{:}L^M \\
&+ k_{\mathrm{d,CD8}}(P^I{:}L^M + P^A{:}L^M + P^P{:}L^M + P^D{:}L^M)
\end{aligned} \tag{36}
$$

3.5 Bound antibodies on cells

The following equations follow the same assumptions described in sections 3.3 and 3.4
PD-L1 bound to tumor cells ($A{:}L^V$) and damaged tumor cells ($A{:}L^M$).

$$\frac{dA{:}L^V}{dt} = -\frac{k_{\text{pfr,T}}}{k_{\text{limit,T}}} T^V A{:}L^V - k_{\text{d,AL}} A{:}L^V - k_{\text{off,AL}} A{:}L^V + k_{\text{on,AL}} A^L L^V/(V_{\text{t}}\epsilon) \quad (37)$$
$$- k_{\text{kill}} A{:}L^V (\sum_{i=1}^{10} E^{D_i})/V_{\text{t}} - f_{\text{ADCC}} \left(\frac{A{:}L^V}{T^V}\right) A{:}L^V$$

$$\frac{dA{:}L^M}{dt} = -k_{\text{d,AL}} A{:}L^M - k_{\text{apop}} A{:}L^M - k_{\text{off,AL}} A{:}L^M + k_{\text{on,AL}} A^L L^M/(V_{\text{t}}\epsilon) \quad (38)$$
$$+ k_{\text{kill}} A{:}L^V (\sum_{i=1}^{10} E^{D_i})/V_{\text{t}} + f_{\text{ADCC}} \left(\frac{A{:}L^V}{T^V}\right) A{:}L^V$$

PD-1 bound to H cells ($A{:}P^H$)

$$\frac{dA{:}P^H}{dt} = -k_{\text{off,AP}} A{:}P^H + k_{\text{on,AP}} A^P P^H/(V_{\text{t}}\epsilon) - k_{\text{d,P}} A{:}P^H - k_{\text{d,H}} A{:}P^H \quad (39)$$

PD-1 bound to inactive ($A{:}P^I$), active ($A{:}P^A$), proliferating ($A{:}P^P$) CD8+ T cells, and CTLs ($A{:}P^D$).

$$\frac{dA{:}P^I}{dt} = -k_{\text{off,AP}} A{:}P^I + k_{\text{on,AP}} A^P P^I/(V_{\text{t}}\epsilon) - k_{\text{d,P}} A{:}P^I - k_{\text{d,CD8}} A{:}P^I \quad (40)$$
$$+ k_{\text{A2Ibasal}} A{:}P^A - f_{\text{activate}} \left(\frac{P^I{:}L^V}{E^I}\right) A{:}P^I$$

$$\frac{dA{:}P^A}{dt} = -k_{\text{off,AP}} A{:}P^A + k_{\text{on,AP}} A^P P^A/(V_{\text{t}}\epsilon) - k_{\text{d,AP}} A{:}P^A - k_{\text{d,CD8}} A{:}P^A \quad (41)$$
$$- k_{\text{A2Ibasal}} A{:}P^A + f_{\text{activate}} \left(\frac{P^I{:}L^V}{E^I}\right) A{:}P^I - k_{pfrCD8} A{:}P^A$$

$$\frac{dA{:}P^P}{dt} = -k_{\text{off,AP}} A{:}P^P + k_{\text{on,AP}} A^P P^P/(V_{\text{t}}\epsilon) - k_{\text{d,AP}} A{:}P^P - k_{\text{d,CD8}} A{:}P^P \quad (42)$$
$$+ k_{pfrCD8} A{:}P^A - k_{pfrCD8} A{:}P^P \frac{E^{P_7}}{\sum_{i=1}^{7} E^{P_i}}$$

$$\frac{dA{:}P^D}{dt} = -k_{\text{off,AP}} A{:}P^D + k_{\text{on,AP}} A^P P^D/(V_{\text{t}}\epsilon) - k_{\text{d,AP}} A{:}P^D - k_{\text{d,CTL}} A{:}P^D \quad (43)$$
$$+ k_{pfrCD8} A{:}P^P \frac{E^{P_7}}{\sum_{i=1}^{7} E^{P_i}}$$

3.6 Bound PD-1:PD-L1 on cells

The following equations follow the same assumptions described in sections 3.3 and 3.4

$$\begin{aligned}\frac{dP^H{:}L^V}{dt} = & -k_{\text{off,PL}}P^H{:}L^V + k_{\text{on,PL}}P^H L^V/(V_{\text{t}}\epsilon) - k_{\text{d,PL}}P^H{:}L^V - k_{\text{d,H}}P^H{:}L^V \\ & - k_{\text{pfr,T}}/k_{\text{limit,T}}T^V P^H{:}L^V - f_{\text{ADCC}}\left(\frac{A{:}L^V}{T^V}\right)P^H{:}L^V\end{aligned} \tag{44}$$

$$\begin{aligned}\frac{dP^H{:}L^M}{dt} = & -k_{\text{off,PL}}P^H{:}L^M + k_{\text{on,PL}}P^H L^M/(V_{\text{t}}\epsilon) - k_{\text{d,PL}}P^H{:}L^M - k_{\text{d,CDH}}P^H{:}L^M \\ & - k_{\text{apop}}P^H{:}L^M + f_{\text{ADCC}}\left(\frac{A{:}L^V}{T^V}\right)P^H{:}L^V\end{aligned} \tag{45}$$

$$\begin{aligned}\frac{dP^I{:}L^V}{dt} = & -k_{\text{off,PL}}P^I{:}L^V + k_{\text{on,PL}}P^I L^V/(V_{\text{t}}\epsilon) - k_{\text{d,PL}}P^I{:}L^V - k_{\text{d,CD8}}P^I{:}L^V \\ & - k_{\text{pfr,T}}/k_{\text{limit,T}}T^V P^I{:}L^V - f_{\text{ADCC}}\left(\frac{A{:}L^V}{T^V}\right)P^I{:}L^V \\ & + k_{\text{A2Ibasal}}P^A{:}L^V - f_{\text{activate}}\left(\frac{P^I{:}L^V}{E^I}\right)P^I{:}L^V\end{aligned} \tag{46}$$

$$\begin{aligned}\frac{dP^I{:}L^M}{dt} = & -k_{\text{off,PL}}P^I{:}L^M + k_{\text{on,PL}}P^I L^M/(V_{\text{t}}\epsilon) - k_{\text{d,PL}}P^I{:}L^M - k_{\text{d,CD8}}P^I{:}L^M \\ & - k_{\text{apop}}P^I{:}L^M + f_{\text{ADCC}}\left(\frac{A{:}L^V}{T^V}\right)P^I{:}L^V \\ & + k_{\text{A2Ibasal}}P^A{:}L^M - f_{\text{activate}}\left(\frac{P^I{:}L^V}{E^I}\right)P^I{:}L^M\end{aligned} \tag{47}$$

$$\begin{aligned}\frac{dP^A{:}L^V}{dt} = & -k_{\text{off,PL}}P^A{:}L^V + k_{\text{on,PL}}P^A L^V/(V_{\text{t}}\epsilon) - k_{\text{d,PL}}P^A{:}L^V - k_{\text{d,CD8}}P^A{:}L^V \\ & - k_{\text{pfr,T}}/k_{\text{limit,T}}T^V P^A{:}L^V - f_{\text{ADCC}}\left(\frac{A{:}L^V}{T^V}\right)P^A{:}L^V \\ & - k_{\text{A2Ibasal}}P^A{:}L^V + f_{\text{activate}}\left(\frac{P^I{:}L^V}{E^I}\right)P^I{:}L^V - k_{\text{pfrCD8}}P^A{:}L^V\end{aligned} \tag{48}$$

$$\begin{aligned}\frac{dP^A{:}L^M}{dt} = & -k_{\text{off,PL}}P^A{:}L^M + k_{\text{on,PL}}P^A L^M/(V_{\text{t}}\epsilon) - k_{\text{d,PL}}P^A{:}L^M - k_{\text{d,CD8}}P^A{:}L^M \\ & - k_{\text{apop}}P^A{:}L^M + f_{\text{ADCC}}\left(\frac{A{:}L^V}{T^V}\right)P^A{:}L^V \\ & - k_{\text{A2Ibasal}}P^A{:}L^M + f_{\text{activate}}\left(\frac{P^I{:}L^V}{E^I}\right)P^I{:}L^M - k_{\text{pfrCD8}}P^A{:}L^M\end{aligned} \tag{49}$$

$$
\begin{aligned}
\frac{dP^P{:}L^V}{dt} = & -k_{\text{off,PL}}P^P{:}L^V + k_{\text{on,PL}}P^P L^V/(V_{\text{t}}\epsilon) - k_{\text{d,PL}}P^P{:}L^V - k_{\text{d,CD8}}P^P{:}L^V \\
& - k_{\text{pfr,T}}/k_{\text{limit,T}}T^V P^P{:}L^V - f_{\text{ADCC}}\left(\frac{A{:}L^V}{T^V}\right)P^P{:}L^V \\
& + k_{\text{pfrCD8}}P^A{:}L^V - k_{\text{pfrCD8}}P^P{:}L^V E^{P_7}/\sum_{i=1}^{7} E^{P_i}
\end{aligned} \tag{50}
$$

$$
\begin{aligned}
\frac{dP^P{:}L^M}{dt} = & -k_{\text{off,PL}}P^P{:}L^M + k_{\text{on,PL}}P^P L^M/(V_{\text{t}}\epsilon) - k_{\text{d,PL}}P^P{:}L^M - k_{\text{d,CD8}}P^P{:}L^M \\
& - k_{\text{apop}}P^P{:}L^M + f_{\text{ADCC}}\left(\frac{A{:}L^V}{T^V}\right)P^P{:}L^V \\
& + k_{\text{pfrCD8}}P^A{:}L^M - k_{\text{pfrCD8}}P^P{:}L^M E^{P_7}/\sum_{i=1}^{7} E^{P_i}
\end{aligned} \tag{51}
$$

$$
\begin{aligned}
\frac{dP^D{:}L^V}{dt} = & -k_{\text{off,PL}}P^D{:}L^V + k_{\text{on,PL}}P^D L^V/(V_{\text{t}}\epsilon) - k_{\text{d,PL}}P^D{:}L^V - k_{\text{d,CTL}}P^D{:}L^V \\
& - k_{\text{pfr,T}}/k_{\text{limit,T}}T^V P^D{:}L^V - f_{\text{ADCC}}\left(\frac{A{:}L^V}{T^V}\right)P^D{:}L^V \\
& + k_{\text{pfrCD8}}P^P{:}L^V E^{P_7}/\sum_{i=1}^{7} E^{P_i}
\end{aligned} \tag{52}
$$

$$
\begin{aligned}
\frac{dP^D{:}L^M}{dt} = & -k_{\text{off,PL}}P^D{:}L^M + k_{\text{on,PL}}P^D L^M/(V_{\text{t}}\epsilon) - k_{\text{d,PL}}P^D{:}L^M - k_{\text{d,CTL}}P^D{:}L^M \\
& - k_{\text{apop}}P^D{:}L^M + f_{\text{ADCC}}\left(\frac{A{:}L^V}{T^V}\right)P^D{:}L^V \\
& + k_{\text{pfrCD8}}P^P{:}L^M E^{P_7}/\sum_{i=1}^{7} E^{P_i}
\end{aligned} \tag{53}
$$

4 Parameter fitting using baseline intra-tumoral T cell concentration data

T cell kinetic parameters were constrained using average concentrations of CD8+ T cells from untreated CT26 tumors in Balb/c mice (Figure 1C, Table S1) that were derived from flow cytometry data. The five CD8+ T cell populations were defined by cell surface

markers and their concentrations were derived as follows:

$$\text{marker concentration} = \frac{\text{viable cell count } (10^6 \text{ per ml})}{\text{percentviable}} \times \text{total events} \times \text{CD45 events}$$
$$\times \text{ CD8 as \%CD45} \times \text{marker as \%CD45}$$

The model set up for the CD8+ T cell stages has the potential to accurately capture the magnitude and speed of T cell expansion after anti-PD-(L)1 treatment, but has a number parameters not readily available in the literature. These include $k_{\text{pfrCD8}}, k_{\text{d,CD8}}, k_{\text{d,CTL}}, E^{\text{activate}}_{\text{max}}$, and $k_{\text{s,I}}$. Our goal is to constrain these parameters so that the model predicts accurate CD8+ T cell concentrations for each stage during control simulations.

The known concentrations are $[E^I]$, $[E^A]$, $[E^P]$, $[E^D]$, and $[E^{D_0}]$ where $[E^P] = \sum_{i=1}^{7} E^{P_i}/V_t$ and $[E^D] = \sum_{i=0}^{10} E^{D_i}/V_t$. See T cell concentration data reference for the specific numbers. Note that these are concentrations, while our original model equations are in amount. We assume that as the tumor grows, the concentrations of CD8+ T cells remains constant. This is true in model simulations due to the $k_{\text{s,I}}V_t$ term in equation 56. We write them now as concentrations for use in this exercise.

$$\frac{d[E^I]}{dt} = f_{\text{influx}}\left(\frac{[P^I{:}L^V]}{[E^I]}\right) - k_{\text{d,CD8}}[E^I] - f_{\text{activate}}\left(\frac{[P^I{:}L^V]}{[E^I]}\right)[E^I] + k_{\text{A2Ibasal}}[E^A] \tag{54}$$

$$f_{\text{activate}}(x) = E^{\text{activate}}_{\text{max}} - \frac{E^{\text{activate}}_{\text{max}}}{1 + \frac{EC50}{x}} \tag{55}$$

$$f_{\text{influx}}(x) = \max\left[0, \frac{E^{\text{influx}}_{\text{max}}}{1 + \frac{EC50}{7.1}} - \frac{E^{\text{influx}}_{\text{max}}}{1 + \frac{EC50}{x}}\right]/V_{\text{t}} + k_{\text{s,I}} \tag{56}$$

$$\frac{d[E^A]}{dt} = f_{\text{activate}}\left(\frac{[P^I{:}L^V]}{[E^I]}\right)[E^I] - k_{\text{d,CD8}}[E^A] - k_{\text{A2Ibasal}}[E^A] - k_{\text{pfr}}[E^A] \tag{57}$$

$$\frac{d[E^{P_1}]}{dt} = 2k_{\text{pfr}}[E^A] - k_{\text{pfr}}[E^{P_1}] - k_{\text{d,CD8}}[E^{P_1}] \tag{58}$$

$$\frac{d[E^{P_i}]}{dt} = 2k_{\text{pfr}}[E^{P_{i-1}}] - k_{\text{pfr}}[E^{P_i}] - k_{\text{d,CD8}}[E^{P_i}], \quad \text{for } i = 2, ..., 7 \tag{59}$$

$$\frac{d[E^{D_{10}}]}{dt} = 2k_{\text{pfr}}[E^{P_7}] - k_{\text{d,CTL}}[E^{D_{10}}] - k_{\text{kill}}[E^{D_{10}}][T^V] \tag{60}$$

$$\frac{d[E^{D_i}]}{dt} = k_{\text{kill}}[E^{D_{i+1}}][T^V] - k_{\text{d,CTL}}[E^{D_i}] - k_{\text{kill}}[E^{D_i}][T^V], \quad \text{for } i = 9, ..., 1 \tag{61}$$

$$\frac{d[E^{D_0}]}{dt} = k_{\text{kill}}[E^{D_1}][T^V] - k_{\text{d,CTL}}[E^{D_0}] \tag{62}$$

Since we are interested in the total number of CD8 cells, and that does not change as they travel through the D_i compartments, we can use $[D] = \sum_{i=0}^{10}[E^{D_i}]$ to simplify equations 60-61 as follows:

$$\frac{d[E^D]}{dt} = 2k_{\text{pfr,CD8}}[P_7] - k_{\text{d,CTL}}[E^D] \tag{63}$$

Step 1: Write steady state concentrations in terms of $[E^I]$
Apply steady state assumptions, so all differential equations = 0.
Let $f_{\text{activate}} = f_{\text{activate}}\left(\frac{[P^I:L^V]}{[E^I]}\right)$ for brevity.
Rearrange equations 57-59, and 63 to get:

$$[E^{P_i}] = \frac{2k_{\text{pfr,CD8}}}{k_{\text{pfr,CD8}} + k_{\text{d,CD8}}}[E^{P_{i-1}}] \text{ for } i = 7, ..., 1 \text{ comes from equations 3 and 4} \tag{64}$$

note:$[E^A] = [E^{P_0}]$

$$[E^{P_i}] = \left(\frac{2k_{\text{pfr,CD8}}}{k_{\text{pfr,CD8}} + k_{\text{d,CD8}}}\right)^i [E^A] \quad \text{comes from repeating previous equation} \tag{65}$$

$$[E^A] = \frac{f_{\text{activate}}}{k_{\text{A2Ibasal}} + k_{\text{pfr,CD8}} + k_{\text{d,CD8}}}[E^I] \quad \text{comes from eq. 57} \tag{66}$$

$$[E^{P_i}] = \left(\frac{2k_{\text{pfr,CD8}}}{k_{\text{pfr,CD8}} + k_{\text{d,CD8}}}\right)^i \frac{f_{\text{activate}}}{k_{\text{A2Ibasal}} + k_{\text{pfr,CD8}} + k_{\text{d,CD8}}}[E^I] \tag{67}$$

$$[E^D] = \frac{2k_{\text{pfr,CD8}}}{k_{\text{d,CTL}}}[E^{P_7}] \quad \text{comes from equation 63} \tag{68}$$

$$[E^D] = \frac{2k_{\text{pfr,CD8}}}{k_{\text{d,CTL}}}\left(\frac{2k_{\text{pfr,CD8}}}{k_{\text{pfr,CD8}} + k_{\text{d,CD8}}}\right)^7 \frac{f}{k_{\text{A2Ibasal}} + k_{\text{pfr,CD8}} + k_{\text{d,CD8}}}[E^I] \tag{69}$$

Step 2: Constrain $k_{\text{d,CD8}}$ in terms of $k_{\text{pfr,CD8}}$ From equation 65 we get:

$$[E^P] = \sum_{i=1}^{7}[E^{P_i}] = \sum_{i=1}^{7}\left(\frac{2k_{\text{pfr,CD8}}}{k_{\text{pfr,CD8}} + k_{\text{d,CD8}}}\right)^i [E^A] \tag{70}$$

$$\frac{[E^P]}{[E^A]} = \sum_{i=1}^{7}\left(\frac{2k_{\text{pfr,CD8}}}{k_{\text{pfr,CD8}} + k_{\text{d,CD8}}}\right)^i = \sum_{i=1}^{7}\left(\frac{2}{1 + \frac{k_{\text{d,CD8}}}{k_{\text{pfr,CD8}}}}\right)^i \tag{71}$$

$\frac{[E^P]}{[E^A]}$ is known, so use a numerical solver to get $k_{\text{d,CD8}} = k_{\text{pfr,CD8}}/2.3581$.
Step 3: Constrain $k_{\text{d,CTL}}$ in terms of $k_{\text{pfr,CD8}}$

From equation 63:

$$k_{\text{d,CTL}} = \frac{2[E^{P_7}]}{[E^D]} k_{\text{pfr,CD8}} \tag{72}$$

$$[E^{P_7}] = [E^P] - \sum_{i=1}^{6} [E^{P_i}] = [E^P] - \sum_{i=1}^{6} \left(\frac{2k_{\text{pfr,CD8}}}{k_{\text{pfr,CD8}} + k_{\text{d,CD8}}} \right)^i [E^A] \tag{73}$$

$$= [E^P] - \sum_{i=1}^{6} \left(\frac{2}{1 + \frac{k_{\text{d,CD8}}}{k_{\text{pfr,CD8}}}} \right)^i [E^A]$$

We solved for $\frac{k_{\text{d,CD8}}}{k_{\text{pfr,CD8}}}$ numerically in step 2. Combine these two equations for the result $k_{\text{d,CTL}} = 11.7403 \times k_{\text{pfr,CD8}}$.

Step 4: Find $k_{\text{s,I}}$ in terms of $[E^I]$ and k_{A2Ibasal}

Use equation 55 with steady state assumptions. Additionally, $\frac{[P^I:L^V]}{[E^I]}$ is at its steady state value so $f_{\text{influx}}\left(\frac{[P^I:L^V]}{[E^I]}\right) = k_{\text{s,I}}$.

$$\frac{d[E^I]}{dt} = 0 = k_{\text{s,I}} - k_{\text{d,CD8}}[E^I] \tag{74}$$

$$- f_{\text{activate}}[E^I] + k_{\text{A2Ibasal}}[E^A]$$

$$k_{\text{s,I}} = k_{\text{d,CD8}}[E^I] + f_{\text{activate}}[E^I] - k_{\text{A2Ibasal}}[E^A] \tag{75}$$

Parameters $k_{\text{s,I}}$ and k_{A2Ibasal} cannot be uniquely identified with high confidence given the limited data available. Thus we fixed k_{A2Ibasal} to the value used in [7] and calculated $k_{\text{s,I}}$ to match.

Step 5: Find lower bound for k_{kill}

We begin by writing all $[E^{D_i}]$ in terms of $[E^{D_0}]$

$$[E^{D_1}] = \frac{k_{\text{d,CTL}}}{k_{\text{kill}}[T^V]}[E^{D_0}] \quad \text{from eq. 62} \tag{76}$$

$$[E^{D_{i+1}}] = \frac{k_{\text{d,CTL}} + k_{\text{kill}}[T^V]}{k_{\text{kill}}[T^V]}[E^{D_i}] \quad \text{from eq. 61} \tag{77}$$

$$[E^{D_{i+1}}] = \left(\frac{k_{\text{d,CTL}}}{k_{\text{kill}}[T^V]} + 1\right)^i \frac{k_{\text{d,CTL}}}{k_{\text{kill}}[T^V]}[E^{D_0}] \quad \text{from eqs. 76,77} \tag{78}$$

$$[E^D] = \sum_{i=0}^{9} \left(\frac{k_{\text{d,CTL}}}{k_{\text{kill}}[T^V]} + 1\right)^i \frac{k_{\text{d,CTL}}}{k_{\text{kill}}[T^V]}[E^{D_0}] + [E^{D_0}] \tag{79}$$

We know $[E^D]$ and $[E^{D_0}]$ and we know $k_{\text{d,CTL}}$ from step 3. Note that $[T^V] = \frac{T^V}{V_t} = \frac{T^V \times C_{TC}}{T^V + T^M} \neq C_{TC}$. The exact ratio of undamaged to damaged tumor cells varies based on many parameters, but in the majority of simulations, almost all tumor cells are undamaged, so we can approximate $[T^V] \approx 0.9999 \times C_{TC}$. Thus, we can find k_{kill} with a numerical solver.

References

[1] Ugo Del Monte. Does the cell number 109 still really fit one gram of tumor tissue? *Cell Cycle*, 8(3):505–506, 2009.

[2] Philip Hahnfeldt, Dipak Panigrahy, Judah Folkman, and Lynn Hlatky. Tumor development under angiogenic signaling: A dynamical theory of tumor growth, treatment response, and postvascular dormancy1. *Cancer Research*, 59(19):4770–4775, 1999.

[3] Stephan Halle, Kirsten Anja Keyser, Felix Rolf Stahl, Andreas Busche, Anja Marquardt, Xiang Zheng, Melanie Galla, Vigo Heissmeyer, Katrin Heller, Jasmin Boelter, Karen Wagner, Yvonne Bischoff, Rieke Martens, Asolina Braun, Kathrin Werth, Alexey Uvarovskii, Harald Kempf, Michael Meyer-Hermann, Ramon Arens, Melanie Kremer, Gerd Sutter, Martin Messerle, and Reinhold Förster. In vivo killing capacity of cytotoxic t cells is limited and involves dynamic interactions and t cell cooperativity. *Immunity*, 44(2):233–245, 2016.

[4] Si-Jie Hao, Yuan Wan, Yi-Qiu Xia, Xin Zou, and Si-Yang Zheng. Size-based separation methods of circulating tumor cells. *Advanced Drug Delivery Reviews*, 125:3–20, 2018.

[5] Angela M. Jarrett, Ernesto A. B. F. Lima, David A. Hormuth, Matthew T. McKenna, Xinzeng Feng, David A. Ekrut, Anna Claudia M. Resende, Amy Brock, and Thomas E. Yankeelov. Mathematical models of tumor cell proliferation: A review of the literature. *Expert Review of Anticancer Therapy*, 18(12):1271–1286, 2018.

[6] Zhe Li, Yingyi Li, Hsuan-Ping Chang, Hsueh-Yuan Chang, Leiming Guo, and Dhaval K. Shah. Effect of size on solid tumor disposition of protein therapeutics. *Drug Metabolism and Disposition*, 47(10):1136–1145, 2019.

[7] Wenlian Qiao, Lin Lin, Carissa Young, Jatin Narula, Fei Hua, Andrew Matteson, Andrea Hooper, Lore Gruenbaum, and Alison Betts. Quantitative systems pharmacology modeling provides insight into inter-mouse variability of anti-ctla4 response. *CPT: Pharmacometrics & Systems Pharmacology*, 2022.

[8] Heesik Yoon, Taeg S. Kim, and Thomas J. Braciale. The cell cycle time of cd8+ t cells responding in vivo is controlled by the type of antigenic stimulus. *PLoS ONE*, 5(11):e15423, 2010.
